# Planar and Pyramidal Pentacoordinate Selenium Atoms

**DOI:** 10.1002/chem.202502525

**Published:** 2025-11-24

**Authors:** Luz Diego, Alejandro Vásquez‐Espinal, Rafael Islas, Gabriel Merino

**Affiliations:** ^1^ Doctorado en Fisicoquímica Molecular Facultad de Ciencias Exactas Universidad Andres Bello Av. República 275 Santiago Chile; ^2^ Química y Farmacia Facultad de Ciencias de la Salud Universidad Arturo Prat Iquique 1100000 Chile; ^3^ Departamento de Ciencias Químicas Facultad de Ciencias Exactas Universidad Andres Bello Av. República 275 Santiago 8370146 Chile; ^4^ Centro de Química Teórica & Computacional (CQT&C) Facultad de Ciencias Exactas Universidad Andres Bello Av. República 275 Santiago 8370146 Chile; ^5^ Departamento de Física Aplicada Centro de Investigación y de Estudios Avanzados Unidad Mérida km. 6 Antigua carretera a Progreso. Apdo. Postal 73, Cordemex Mérida México

**Keywords:** chemical bond, hypercoordination, planarity, selenium

## Abstract

A systematic computational exploration of the dianionic systems SeM_5_X_5_^2−^ (M = Li─Cs; X = F─I) was conducted to evaluate the viability of planar pentacoordinate selenium (ppSe). Of the twenty combinations considered, 10 adopt planar or pyramidal structures, with four corresponding to true global minima containing a ppSe center. Bonding analysis and magnetic response calculations show that the stabilization of ppSe arrangements in these dianions results mainly from geometric confinement and strong electrostatic interactions between selenium and the surrounding alkali metal and halogen atoms. Unlike planar hypercoordinate systems stabilized by delocalized bonding or aromaticity, the dianions studied here display highly localized electronic structures, expanding the scope of nonclassical bonding motifs in main‐group chemistry.

## Graphical abstract

Planar (D_5h_) and pyramidal (C_5v_) dianionic clusters, SeM_5_X_5_^2−^ (M = LiK; X = FBr), showcase pentacoordinate selenium atoms stabilized by geometric confinement and electrostatic interactions with surrounding alkali and halogen atoms.

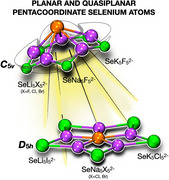

## 1 Introduction

The exploration of nonstandard coordination numbers in carbon has reshaped the description of chemical bonding, challenging traditional paradigms. A notable example is the planar tetracoordinate carbon (ptC), proposed theoretically in 1968,^[^ ^1^ ^]^ revisited in 1970,^[^ ^2^ ^]^ and experimentally verified in 1977,^[^ ^3^ ^]^ with broader recognition in 1979.^[^ ^4^ ^]^ Initial stabilization strategies relied on electronic and steric modifications,^[^ ^5^ ^]^ until Schleyer and Boldyrev showed that 18‐valence‐electron (18‐ve) pentaatomic systems could stabilize ptC species.^[^ ^6^ ^]^ Although the 18‐ve rule remains a valuable design principle,^[^ ^7^, ^8^, ^9^, ^10^, ^11^ ^]^ it is not absolute; ptC species with different electron counts have been identified in the gas‐phase,^[^ ^12^, ^13^, ^14^, ^15^, ^16^ ^]^ synthesized,^[^ ^17^, ^18^ ^]^ or proposed as viable systems.^[^ ^19^, ^20^, ^21^, ^22^ ^]^ These advances established both robust theoretical predictions and experimental confirmations of diverse ptC‐containing molecules.^[^ ^23^, ^24^, ^25^, ^26^, ^27^ ^]^

This framework was later extended to planar penta‐^[^ ^28^, ^29^ ^]^ and hexacoordinate carbon,^[^ ^30^, ^31^ ^]^ as well as to other main‐group elements,^[^ ^32^, ^33^, ^34^ ^]^ expanding the accessible coordination landscape. For example, planar pentacoordinate *s*‐block elements such as lithium, beryllium, and magnesium have been theoretically designed, with stability arising mainly from σ‐type multicenter bonding rather than π‐delocalization.^[^ ^35^, ^36^ ^]^ In contrast, planar hypercoordination in electronegative *p*‐block elements such as nitrogen, oxygen, and fluorine faces additional challenges due to their localized bonding patterns. Nevertheless, suitably constrained geometric and electronic frameworks, often guided by the 18‐ve rule, have enabled the design of ptN,^[^ ^11^ ^]^ ptO,^[^ ^37^ ^]^ and ptF species.^[^ ^38^ ^]^

Our study on ptOs^[^ ^37^ ^]^ shows that appropriate electronic and structural conditions can stabilize these species despite the usual bonding preferences of oxygen. This raised the question of whether heavier group 16 elements can also adopt planar hypercoordinate environments. Density functional theory (DFT) and molecular dynamics simulations predict viable B_4_S monolayers with planar tetracoordinate sulfur,^[^ ^39^ ^]^ while planar pentacoordinate sulfur corresponds to a local minimum in hydrometallic complexes such as SAg_5_H_5_^+^ and SAu_5_H_5_^+^,^[^ ^40^ ^]^ and appears as a global minimum in the Mg_5_S_6_^2−^ cluster.^[^ ^41^ ^]^ Experimentally, tetracoordinate sulfur was found in MCOF‐89 within [Mn_4_(μ_4_–S)],^[^ ^42^ ^]^ and structurally confirmed planar pentacoordinate sulfur is present in [Ni_5_S(SBu^t^)_5_]^−^.^[^ ^43^ ^]^ For selenium, X‐ray diffraction has revealed a nearly perfect square‐planar Se(II) center, as in a Se(Se_4_) core complex,^[^ ^44^ ^]^ while Rufino–Felipe et al. reassigned some species as square‐planar Se(II) derivatives of R_2_P(Se)NH(Se)PR_2_ ligands.^[^ ^45^ ^]^ Most recently, *cis*‐Se(dptu)_2_Cl_2_ and Te(dptu)_2_Cl_2_ have been reported to contain square‐planar Se(II) and Te(II) centers, respectively.^[^ ^46^ ^]^

An effective strategy for stabilizing planar hypercoordinate centers involves dual coordination spheres, with an inner sphere of electropositive metals (e.g., Li, Be, or transition metals) and an outer sphere of electronegative ligands. This design has supported the theoretical prediction and, in some cases, experimental realization of planar pentacoordinate (ppE)^[^ ^47^, ^48^, ^49^ ^]^ and hexacoordinate (phE)^[^ ^50^, ^51^ ^]^ atom species with heavier main‐group central atoms. Examples include GaBe_6_Au_6_^+^,^[^ ^52^ ^]^ EBe_3_Li_3_H_6_ (E = P, As),^[^ ^53^ ^]^ and EM_5_H_5_ (M = Ag, Au, Pd, Pt; E = Si, Ge, P, S).^[^ ^40^ ^]^ Planar pentacoordinate halogens, such as Li_5_Cl_6_^−^ and Li_5_Br_6_^−^, further exemplify the dominant role of electrostatic stabilization.^[^ ^54^, ^55^, ^56^ ^]^ In this context, we now apply this approach, combining dual coordination spheres with targeted electronic design, to the prediction and stabilization of planar pentacoordinate selenium (ppSe).

## 2 Methodology

We investigated the dianionic systems SeM_5_X_5_^2−^ (M = Li─Cs; X = F─I). Initial geometry optimizations for all 20 combinations were performed under *D*_5_ *_h_* and *C*_5_ *_v_* symmetries at the PBE0^[^ ^57^ ^]^‐D3^[^ ^58^ ^]^/def2‐TZVP^[^ ^59^ ^]^ level to evaluate the feasibility of selenium pentacoordination. Ten systems retained the *C*_5_ *_v_* and *D*_5_ *_h_* symmetries. To further explore the potential energy surfaces (PESs) of these 10 systems, the Kick stochastic algorithm implemented in GLOMOS was employed^[^ ^60^, ^61^ ^]^ at the PBE0/SDDAll level.^[^ ^62^, ^63^ ^]^ Isomers within 30 kcal·mol^−1^ of the global minimum were re‐optimized and characterized at the PBE0‐D3/def2‐TZVP level. Harmonic frequency analyses confirmed all re‐optimized structures as true minima.

Single‐point energies were computed at the CCSD(T)^[^ ^64^ ^]^ level using a combination of cc‐pVTZ and cc‐pVTZ‐PP basis sets,^[^ ^65^, ^66^, ^67^ ^]^ with the latter applied to Se, Br, I, and Rb. Zero‐point energy corrections were derived from PBE0‐D3/def2‐TZVP frequencies. Wavefunction stability^[^ ^68^ ^]^ and *T*_1_ diagnostics^[^ ^69^ ^]^ confirmed negligible multireference character for both planar and pyramidal selenium‐containing minima. To evaluate spin‐state preferences, all singlet minima were recalculated as triplets.

Vertical detachment energies (VDE) for the dianionic species were computed using the third‐order partial renormalization (P3+) method^[^ ^70^ ^]^ within electron propagator theory (EPT).^[^ ^71^ ^]^ This approach is valid when the Dyson pole strengths exceed 0.85, a condition satisfied in all cases. These calculations employed the aug‐cc‐pVTZ basis set.^[^ ^72^, ^73^, ^74^ ^]^ All quantum chemical calculations were carried out using Gaussian 16.^[^ ^75^ ^]^

Chemical bonding was examined using Natural Population Analysis (NPA)^[^ ^76^ ^]^ and Wiberg Bond Indices (WBI)^[^ ^77^ ^]^ via NBO 7.0.^[^ ^78^ ^]^ Canonical molecular orbitals and Adaptive Natural Density Partitioning (AdNDP)^[^ ^79^ ^]^ analyses were performed at the PBE0‐D3/def2‐TZVP level using Multiwfn.^[^ ^80^ ^]^

To examine the factors that determine the stability and geometry of the molecular systems, Energy Decomposition Analysis (EDA)^[^ ^81^, ^82^ ^]^ was employed. This method quantifies the individual energetic contributions (electrostatic, orbital, and Pauli repulsion) within predefined molecular fragments, clarifying the balance of forces responsible for the bonding patterns. All calculations were carried out with ADF 2024,^[^ ^83^ ^]^ using the PBE0‐D3/TZ2P^[^ ^84^ ^]^‐ZORA^[^ ^85^ ^]^ level, which provides a consistent framework for evaluating electronic structures and interaction profiles. The Isomerization Energy Decomposition Analysis (IEDA),^[^ ^86^ ^]^ originally developed to identify the energetic factors stabilizing one isomer relative to another, was employed here to compare a local minimum and a stationary point. In this case, the resulting value corresponds to the planarization energy, and the term IEDA was retained for consistency with our previous studies.

Interatomic interaction energies (*V*_int_) were decomposed within the Bader theory through the Interaction Quantum Atoms (IQA) method.^[^ ^87^ ^]^ *V*_int_ was partitioned between atomic basins into a classical Coulombic term (*V*_C_), representing electrostatic interactions, and an exchange‐correlation term (*V*_X_), accounting for quantum effects such as Pauli repulsion and electron correlation. These contributions correlated with the ionic and covalent components of bonding, respectively. IQA analyses were carried out with AIMAll^[^ ^88^ ^]^ using wavefunctions obtained at the PBE0‐D3/def2‐TZVP level.

Magnetic response properties were examined through induced current densities (**J** ^ind^) computed with GIMIC^[^ ^89^ ^]^ at the BHandHLYP^[^ ^90^ ^]^/def2‐TZVP level and visualized in Paraview 5.10.0.^[^ ^91^, ^92^ ^]^ Ring current strengths were quantified by integrating **J** ^ind^ over planes perpendicular to the molecular plane using the 2D Gauss–Lobatto algorithm in GIMIC.^[^ ^89^, ^93^ ^]^ Induced magnetic fields (**B** ^ind^),^[^ ^94^ ^]^ with emphasis on the perpendicular component (***B*** ^ind^_z_ ≡ NICS_zz_),^[^ ^95^ ^]^ were calculated with the screening tensor formalism implemented in ORCA.^[^ ^96^, ^97^ ^]^

The Electron Localization Function (ELF)^[^ ^98^ ^]^ was employed as an auxiliary descriptor to evaluate the presence and extent of electron delocalization in the studied molecular systems. ELF analyses were performed with Multiwfn^[^ ^80^ ^]^ at the PBE0‐D3/def2‐TZVP level, using the converged wavefunctions obtained from the optimized structures. This approach enabled the visualization and quantification of localized versus delocalized electron domains, providing a real‐space description of bonding patterns. In particular, the analysis focused on identifying continuous basins associated with in‐plane electron density, which can indicate multicenter delocalization.

The kinetic stability of the clusters was evaluated through Born–Oppenheimer molecular dynamics (BOMD)^[^ ^99^ ^]^ simulations using deMon2k.^[^ ^100^ ^]^ Each simulation was performed for 30 ps with a timestep of 1 fs. Temperature was controlled with a Nosé–Hoover chain thermostat.^[^ ^101^, ^102^ ^]^ The hybrid PBE0 functional was employed in conjunction with the DZVP basis set for all systems.^[^ ^103^ ^]^

## 3 Structure and Energy

**Figure 1:**
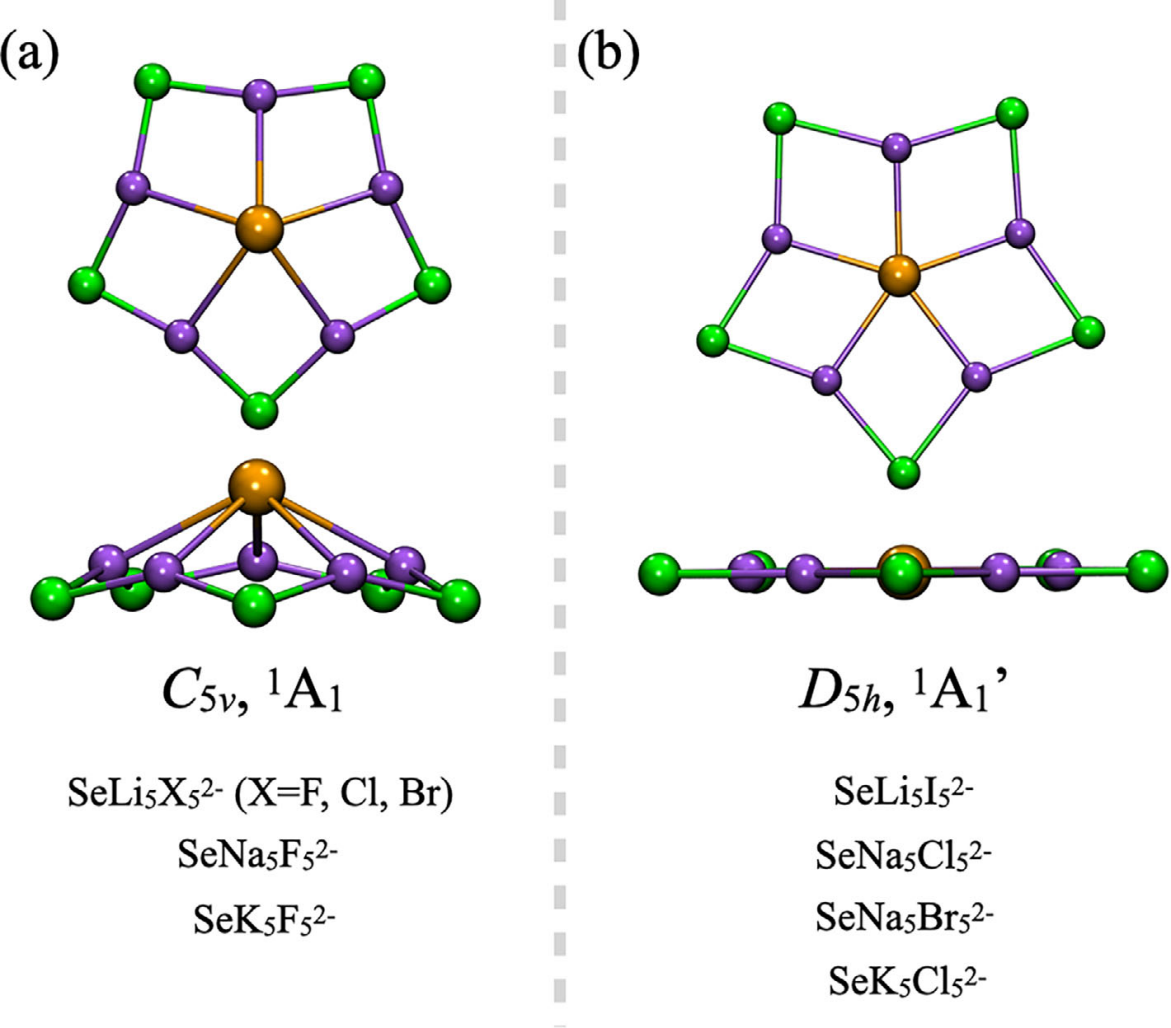
Structures featuring a) a pyramidal pentacoordinate selenium atom with *C*_5_ *_v_* symmetry and b) a planar pentacoordinate selenium (ppSe) atom with *D*_5_ *_h_* symmetry. Point group symmetries and spectroscopic states are indicated below each structure. Orange spheres represent selenium atoms, purple spheres correspond to alkali metals (M = Li─Rb), and green spheres denote halogens (X = F─I).

Among the twenty SeM_5_X_5_^2−^ (M = Li─Rb; X = F─I) combinations, 10 structures with pentacoordinate selenium and *C*_5_ *_v_* or *D*_5_ *_h_* symmetries were identified. The remaining systems, although also pentacoordinate, relaxed to lower symmetry with *C*_s_ or *C*_1_ point groups (see Table S1). Exploration of the PESs located five global minima with *C*_5_ *_v_* pyramidal pentacoordinate selenium centers in SeLi_5_X_5_^2−^ (X = F─Br), SeNa_5_F_5_^2−^, and SeK_5_F_5_^2−^ (Figure 1a). Four additional combinations with ppSe and *D*_5_ *_h_* symmetry were found for SeLi_5_I_5_^2−^, SeNa_5_Cl_5_^2−^, SeNa_5_Br_5_^2−^, and SeK_5_Cl_5_^2−^ (Figure 1b). In SeRb_5_F_5_^2−^, a *C*_5_ *_v_* local minimum was located 1.6 kcal·mol^−1^ above the global minimum.

Triplet‐state isomers were generally less stable than the singlet counterparts, with relative energies between 10 and 91 kcal·mol^−1^ above the global minima. The only exception was SeRb_5_F_5_^2−^, whose triplet state was 1.3 kcal·mol^−1^ above the global minimum.

All isomers exhibited *T*_1_ values below 0.02, indicating that their electronic structures were well‐described by single‐reference methods. The VDE values calculated with the P3+ method showed that the stability of the dianionic systems depends on both the halogen and the metal cation (Table 1). Systems containing fluorine displayed low VDEs, consistent with weak binding of the second electron. In contrast, systems with heavier halogens (Cl, Br, I) showed higher VDEs, which can be attributed to their greater polarizability. Smaller cations such as Li stabilized the system more effectively than larger ones such as K. So, the results confirm that the dianions considered are thermodynamically and electronically stable.

**Table 1:** Lowest vibrational frequency (*ν*_min_, in cm^−1^), HOMO–LUMO gap (gap, in eV), bond distances (*r*, in Å), natural population analysis (NPA) charges (*q*, in |e|), Wiberg bond indices (WBIs) and vertical detachment energies (VDE, in eV) calculated with the P3+ method for SeLi_5_X_5_^2−^ (X = F, Cl, Br, I), SeNa_5_X_5_^2−^ (X = F, Cl, Br), and SeK_5_X_5_^2−^ (X = F, Cl) with *C*_5_ *_v_* and *D*_5_ *_h_* symmetry.

	PG	*ν*_min_	gap	$r_{\mathrm{Se}-M}$	$r_{M-M}$	$r_{E-M}$	q[Se]	q[M]	q[X]	$WBI_{\mathrm{Se}-M}$	$WBI_{M-M}$	$WBI_{M-X}$	VDE_P3+_
SeLi_5_F_5_^2−^	*C*_5_ *_v_*	62	4.43	2.59	2.51	1.78	−1.74	+0.88	−0.93	0.096	0.002	0.061	0.04
SeLi_5_Cl_5_^2−^	*C*_5_ *_v_*	29	5.12	2.49	2.76	2.30	−1.69	+0.81	−0.87	0.112	0.003	0.114	1.24
SeLi_5_Br_5_^2−^	*C*_5_ *_v_*	17	5.20	2.48	2.85	2.47	−1.69	+0.80	−0.86	0.110	0.003	0.123	1.57
SeLi_5_I_5_^2−^	*D*_5_ *_h_*	9	5.35	2.47	2.91	2.69	−1.70	+0.78	−0.84	0.106	0.003	0.149	2.0
SeNa_5_F_5_^2−^	*C*_5_ *_v_*	34	3.45	2.90	3.03	2.15	−1.78	+0.90	−0.95	0.084	0.001	0.049	0.01
SeNa_5_Cl_5_^2−^	*D*_5_ *_h_*	5	5.18	2.82	3.31	2.65	−1.77	+0.87	−0.92	0.086	0.001	0.078	1.04
SeNa_5_Br_5_^2−^	*D*_5_ *_h_*	10	5.20	2.83	3.30	2.81	−1.76	+0.86	−0.91	0.089	0.001	0.084	1.41
SeK_5_F_5_^2−^	*C*_5_ *_v_*	15	2.74	3.28	3.60	2.51	−1.82	+0.92	−0.96	0.070	0.001	0.039	0.24
SeK_5_Cl_5_^2−^	*D*_5_ *_h_*	4	4.76	3.25	3.82	3.03	−1.81	+0.91	−0.95	0.071	0.001	0.053	0.94

Structural and energetic data for all global and relevant local minima are provided in Figures S1–S10. To further confirm the stability of the *D*_5_ *_h_* global minima, additional geometry optimizations were carried out using various DFT functionals and the aug‐cc‐pVTZ basis set (aug‐cc‐pVTZ‐PP for Se, Br, or I‐containing systems). In all cases, the ppSe structures were confirmed as true minima, with no imaginary vibrational frequencies (see Table S2).

For the SeLi_5_X_5_^2−^ series (X = F, Cl, Br, I), the Se─Li distance decreased progressively from 2.59 (F) to 2.47 Å (I), while the Li─Li and Li─X distances increased from 2.51 to 2.91 Å and from 1.78 to 2.69 Å, respectively, as the halogen size increased. This trend maintains the selenium atom within the molecular plane, as occurs in SeLi_5_I_5_^2−^, which exhibits *D*_5_ *_h_* symmetry. A similar pattern was found in the SeNa_5_X_5_^2−^ series (X = F, Cl, Br) and SeK_5_X_5_^2−^ (X = F, Cl), favoring a ppSe in SeNa_5_Cl_5_^2−^, SeNa_5_Br_5_^2−^, and SeK_5_Cl_5_^2−^. The corresponding Se─M, M─M, and M─X distances are summarized in Table 1, confirming that the cavity required to preserve the selenium atom in the plane remains adequate even as the metal size increases.

Comparison with covalent radii proposed by Pyykkö and Atsumi^[^ ^104^ ^]^ indicated that the Se─Li bond lengths in SeLi_5_X_5_^2−^ (X = Cl, Br, I) fall within the expected covalent range (sum of covalent radii ≈ 2.49 Å). The exception is SeLi_5_F_5_^2−^, where the Se─Li distance (2.59 Å) slightly exceeds this value. In contrast, the Se─M distances in SeNa_5_X_5_^2−^ (X = F, Cl, Br) and SeK_5_X_5_^2−^ (X = F, Cl) exceed the respective covalent radius sums (2.64 Å for Na─Se and 2.78 Å for K─Se), consistent with weaker, predominantly electrostatic interactions.

NPA charges showed that in all global minima with planar or pyramidal pentacoordination, both the Se atom and the X atoms carried negative charges, balanced by five positively charged metal atoms. As X increased in size, its negative charge decreased, indicating minor charge redistribution within the system. WBI supported these trends. Se─M and M─X interactions displayed low WBI values, ranging from 0.112 to 0.070 and from 0.149 to 0.034, respectively, indicating limited covalent character and the predominance of weak or ionic interactions. Extremely low WBI values for M─M pairs (<0.003) indicated negligible or repulsive interactions between metal atoms.

Table S1 shows a clear trend: highly symmetric (*C*_5_ *_v_* or *D*_5_ *_h_*) structures are obtained preferentially for lighter alkali metals combined with smaller halogens (upper left of the table), whereas heavier cations and larger halides (lower right) lead to symmetry breaking (*C_s_* or *C*_1_). This diagonal separation can be explained by the increasing mismatch between atomic radii: larger alkali metals and halogens reduce the ability of the five ligands to maintain a symmetric planar environment around selenium, favoring distortions. Within the symmetric cases, there is also a systematic decrease in the lowest vibrational frequency as the size of M and E increases, consistent with a weakening of geometric confinement. These trends indicate that atomic size and the resulting balance between electrostatic attraction and steric repulsion are the primary factors controlling the preservation of symmetry in these dianions.

## 4 Bonding

**Figure 2:**
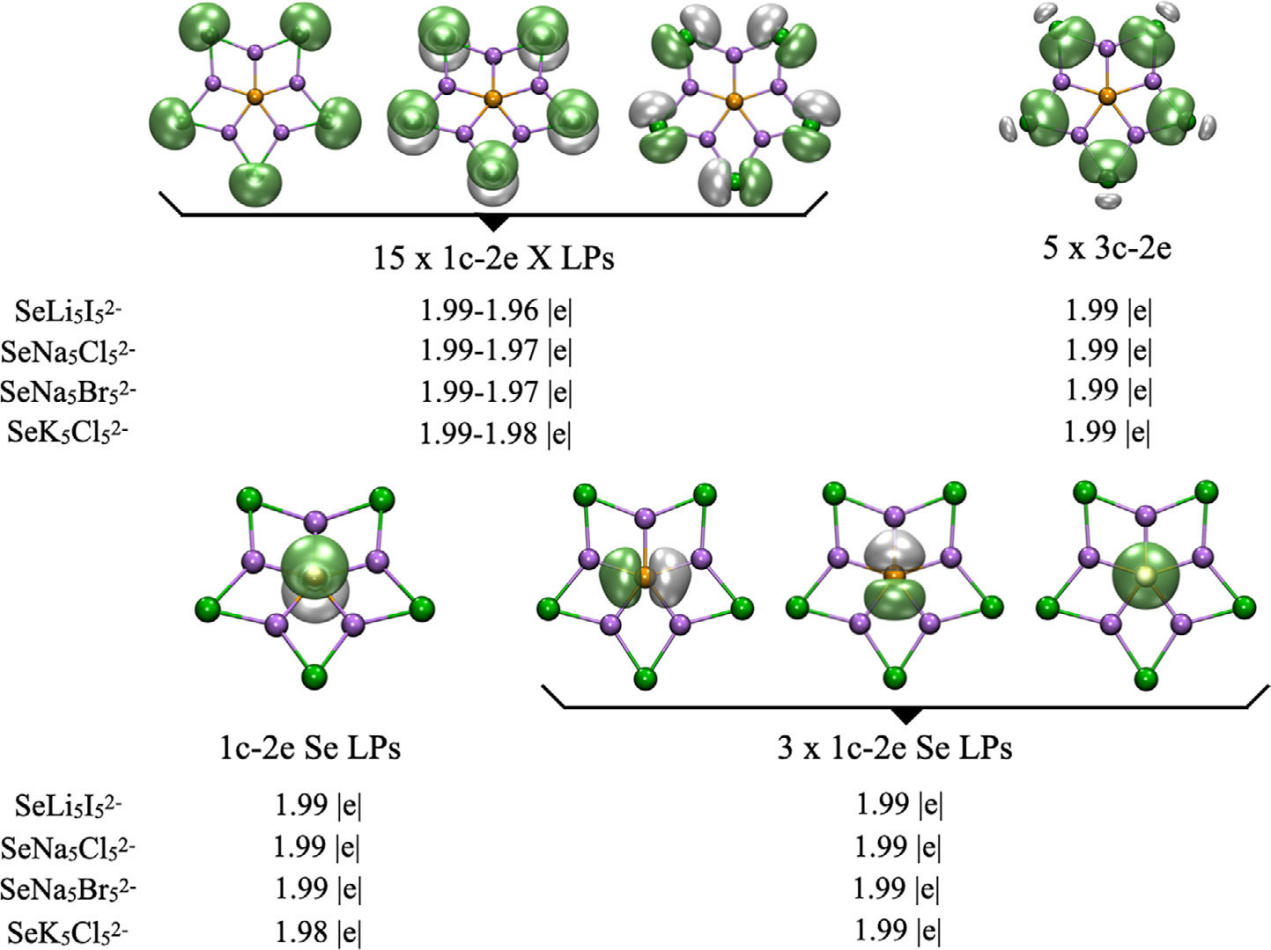
Bonding patterns obtained from AdNDP analysis, showing occupancy numbers (ON, in |e|), computed at the PBE0‐D3/def2‐TZVP level.

The AdNDP analysis (Figure 2) identified fifteen lone pairs localized in the *s* and *p* orbitals of the halogens, all with occupancy numbers (ONs) greater than 1.96 |e|, five 3c–2e σ bonds, and one lone pair in the *p*_z_ orbital of selenium with an ON of 1.99 |e|. Additionally, three lone pairs (1c–2e) were localized on the selenium atom. Although these selenium‐based orbitals can formally be described as 6c–2e multicenter bonds involving Se and the five metal atoms, the electron density remained essentially confined to Se. The multicenter representation artificially inflates the occupation numbers without indicating genuine delocalization. Therefore, the 1c–2e description more accurately reflects the localized lone‐pair character on selenium and the absence of significant bonding interactions with the surrounding metals.

When an interaction has a significant covalent component, any localization scheme, including WBI, is valid. In contrast, small WBI values between two atoms may indicate either a negligible covalent contribution or that the overlap is minimal, with the interaction dominated by electrostatic interactions. To confirm this, analyses of orbitals and bond orders are always complemented by two energy decomposition approaches.

We first carried out an energy decomposition analysis (EDA) à la Morokuma–Ziegler–Rauk. The EDA results (Table S3) showed that bonding in SeM_5_X_5_^2−^​ clusters is predominantly ionic, with the electrostatic term (Δ*V*_elstat_) contributing 75–90% of the stabilizing interactions. This confirms the essentially ionic nature of these systems. Notably, the relative electrostatic contribution increased from Li to Na and K, while the orbital term decreased, indicating that the bonding becomes more ionic with larger alkali metals. Within the Li series, the orbital contribution slightly increased from F to I, suggesting a modest rise in covalent character with heavier halogens. The Pauli repulsion was also systematically higher in the planar structures than in the pyramidal forms, hinting at its possible role in destabilizing the planar arrangement. However, despite the dominant electrostatic term, electrostatic interactions alone did not determine the preferred geometry.

To understand the origin of the geometric preference, we performed an isomerization energy decomposition analysis (IEDA) on the SeLi_5_X_5_^2−^ (X = F, Cl, Br), SeNa_5_F_5_^2−^, and SeK_5_F_5_^2−^ clusters. In this approach, the isomerization energy (Δ*E*_iso_), defined as the energy difference between the pyramidal and planar structures, formally corresponds to a planarization energy. However, the term isomerization energy is retained to maintain consistency with the original IEDA methodology. Δ*E*_iso_ was partitioned into the distortion energy (Δ*E*_dist_), which quantifies the cost of distorting the M_5_X_5_ fragment from *D*_5_ *_h_* to *C*_5_ *_v_*, and the change in interaction energy (ΔΔ*E*_int_), which reflects the difference in Se^2−^─M_5_X_5_ interactions between the two symmetries. Negative values indicate a preference for the *C*_5_ *_v_* form, while positive values favor the *D*_5_ *_h_* structure. The term ΔΔ*E*_int_ was further divided into orbital (ΔΔ*E*_oi_), electrostatic (ΔΔ*V*_elstat_), Pauli repulsion (ΔΔ*E*_Pauli_), and dispersion (ΔΔ*E*_disp_) contributions (Table 2).

**Table 2:** Isomerization energy decomposition analysis (IEDA) at the PBE0‐D3/TZ2P‐ZORA//PBE0‐D3/aug‐cc‐pVTZ level for the SeM_5_X_5_^2−^ clusters. Energy values are given in kcal·mol^−1^.

System	Δ*E*_iso_	Δ*E*_dist_	ΔΔ*E*_int_	ΔΔ*E*_oi_	ΔΔ*V*_elstat_	ΔΔ*E*_Pauli_	ΔΔ*E*_disp_
SeLi_5_F_5_^2−^	−25.1	2.5	−27.6	12.2	47.0	−86.9	0.2
SeLi_5_Cl_5_^2−^	−1.5	5.4	−7.0	−2.6	12.0	−16.5	0.1
SeLi_5_Br_5_^2−^	−0.2	2.0	−2.2	−1.5	4.0	−4.7	0.0
SeNa_5_F_5_^2−^	−7.5	9.6	−17.2	−1.7	30.0	−45.6	0.2
SeK_5_F_5_^2−^	−2.2	3.2	−5.5	2.3	20.2	−28.2	0.1

In all systems, the distortion energy favored the *D*_5_ *_h_* geometry, but this effect was consistently outweighed by the ΔΔ*E*_int_ term, which drove the preference for the pyramidal arrangement. Within ΔΔ*E*_int_, the Pauli repulsion was systematically larger in the planar structure and emerged as the decisive factor stabilizing the pyramidal form. The largest Pauli repulsion differences occurred in SeLi_5_F_5_^2−^ and SeNa_5_F_5_^2−^, which also showed the most negative isomerization energies, indicating a clear correlation between the magnitude of the Pauli term and the stability of the pyramidal structure. By contrast, the electrostatic interaction was consistently more favorable in the planar form, especially in the Li and Na compounds, but could not overcome the stronger Pauli repulsion of this form. Orbital contributions showed no consistent trend, favoring the planar structure in SeLi_5_F_5_^2−^ and SeK_5_F_5_^2−^, but the pyramidal structure in SeLi_5_Cl_5_^2−^, SeLi_5_Br_5_^2−^, and SeNa_5_F_5_^2−^. However, they were much smaller in magnitude than the electrostatic and Pauli terms, indicating that they play only a secondary role. Finally, dispersion contributions were negligible in all cases, showing that dispersion effects remain essentially unchanged between the two geometries.

While the EDA provides valuable insight into the interactions between molecular fragments, it does not give a detailed picture of the energy distribution at the atomic level. To complement this analysis and gain a more rigorous understanding of the bonding within the molecule, we next employ the Interacting Quantum Atoms (IQA) approach, which decomposes the total energy into atom‐by‐atom contributions based on Bader theory. Table 3 presents the IQA results for the global minima of species with pentacoordinate selenium in planar and pyramidal geometries. Se─M interactions were consistently attractive, ranging from −210.1 to −145.0 kcal·mol^−1^, and decreased in magnitude as the metal size increased. These interactions were dominated by the Coulombic term, with minor contributions from exchange (−17.2 to −8.9 kcal·mol^−1^), indicating predominantly ionic character. M─M interactions were repulsive in all cases (104.9 to 62.4 kcal·mol^−1^), governed mainly by Coulombic repulsion with negligible exchange stabilization. Similarly, the M─X interactions were attractive (−135.8 to −84.2 kcal·mol^−1^) and decreased with increasing size of X, also exhibiting primarily Coulombic character. Overall, Se─M and M─X interactions were essentially ionic, supporting the structural stabilization of ppSe through electrostatic effects.

**Table 3:** Interatomic interaction energy components from IQA analysis (in kcal·mol^−1^) for SeLi_5_X_5_^2−^ (X = F, Cl, Br, I), SeNa_5_X_5_^2−^ (X = F, Cl, Br), and SeK_5_X_5_^2−^ (X = F, Cl) with *C*_5_ *_v_* and *D*_5_ *_h_* symmetry, computed at the PBE0‐D3/def2‐TZVP level. Δ*E*_IQA_ represents the integration error. The reported values include the total interatomic interaction energy, the Coulomb component, and the exchange–correlation component.

	SeLi_5_F_5_^2−^ [*C*_5_ *_v_*]	SeLi_5_Cl_5_^2−^ [*C*_5v_]	SeLi_5_Br_5_^2−^ [*C*_5_ *_v_*]	SeLi_5_I_5_^2−^ [*D*_5_ *_h_*]	SeNa_5_F_5_^2−^ [*C*_5_ *_v_*]	SeNa_5_Cl_5_^2−^ [*D*_5_ *_h_*]	SeNa_5_Br_5_^2−^ [*D*_5_ *_h_*]	SeK_5_F_5_^2−^ [*C*_5_ *_v_*]	SeK_5_Cl_5_^2−^ [*D*_5_ *_h_*]
$\Delta E_{\mathrm{IQA}}$	0.0	0.0	0.0	0.0	0.0	0.0	0.0	0.0	0.0
$V_{\mathrm{IQA}}^{\mathrm{Int}}\left(Se-M\right)$	−206.5	−210.1	−208.6	−207.2	−175.0	−175.4	−173.8	−155.1	−154.3
$V_{C}^{\mathrm{Int}}\left(Se-M\right)$	−197.6	−199.1	−197.3	−195.7	−161.7	−160.7	−159.2	−138.5	−137.2
$V_{\mathrm{XC}}^{\mathrm{Int}}\left(Se-M\right)$	−8.9	−11.0	−11.3	−11.5	−13.3	−14.7	−14.6	−16.6	−17.1
$V_{\mathrm{IQA}}^{\mathrm{Int}}\left(M-M\right)$	104.9	93.1	90.2	87.2	82.7	73.9	72.5	68.2	62.4
$V_{C}^{\mathrm{Int}}\left(M-M\right)$	104.9	93.2	90.3	87.3	82.9	74.0	72.6	68.6	62.7
$V_{XC}^{Int}\left(M-M\right)$	0.0	−0.1	−0.1	−0.1	−0.2	−0.1	−0.1	−0.5	−0.3
$V_{IQA}^{Int}\left(X-M\right)$	−166.3	−131.9	−124.0	−114.4	−139.7	−112.8	−106.4	−124.4	−100.9
$V_{C}^{\mathrm{Int}}\left(X-M\right)$	−150.9	−121.0	−114.1	−105.3	−123.0	−100.0	−94.3	−105.0	−85.9
$V_{\mathrm{XC}}^{\mathrm{Int}}\left(X-M\right)$	−15.4	−10.9	−9.9	−9.1	−16.7	−12.8	−12.1	−19.4	−15.0

Although bonding analyses indicated that the interactions in systems with pentacoordinate selenium are predominantly ionic, the (non)aromatic character was assessed through magnetic ring current analysis. This analysis focused on systems in which the ppSe structure corresponded to the global minimum to determine whether stabilization involved electron delocalization at the molecular level. The absence of significant ring currents would indicate ionic behavior, whereas their presence would suggest an aromatic contribution.

**Figure 3:**
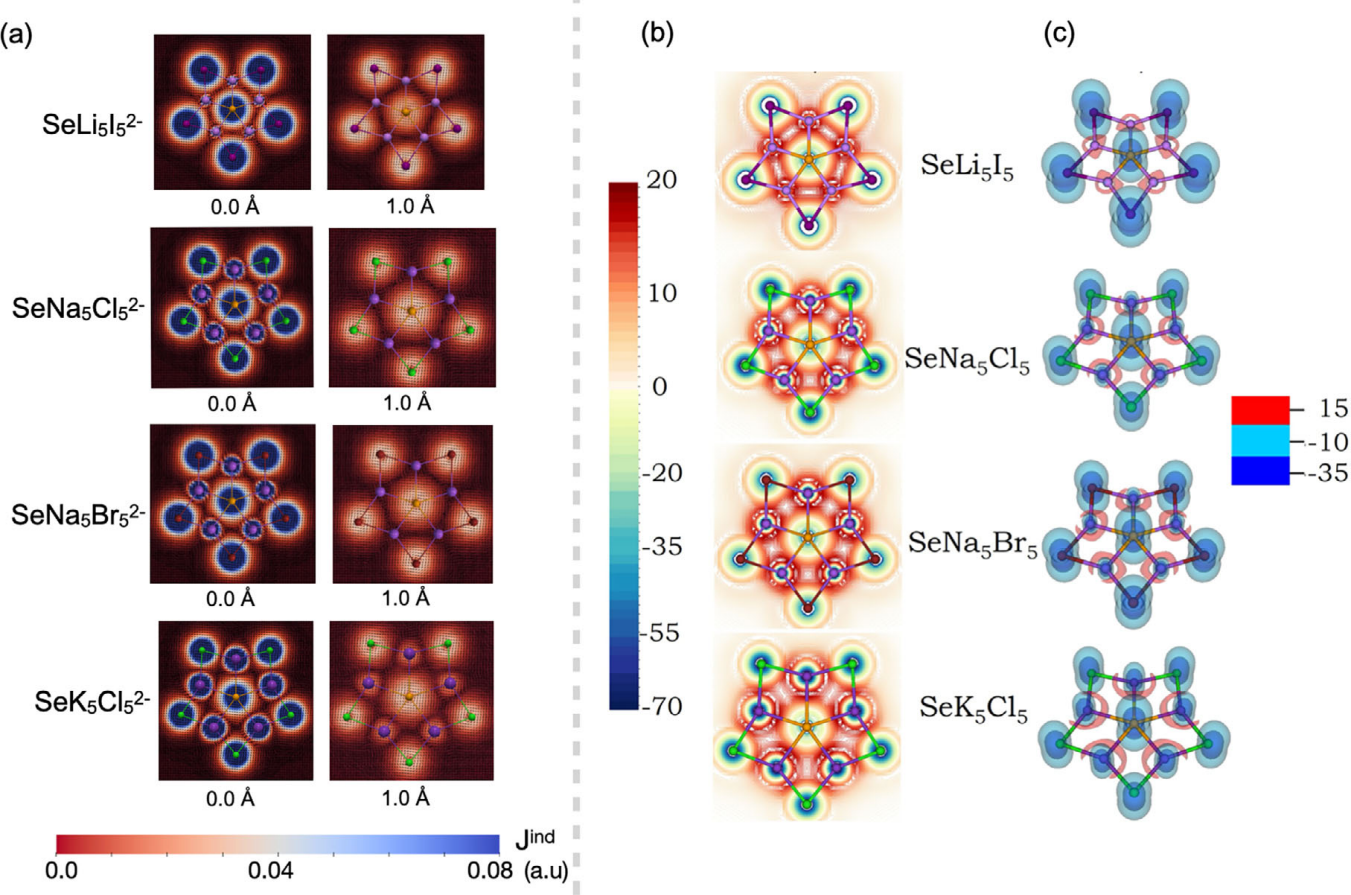
a) Vector field plots of the induced current density (**J** ^ind^) in the molecular plane (0.0 Å) and at 1.0 Å above the plane for SeLi_5_I_5_^2−^, SeNa_5_Cl_5_^2−^, SeNa_5_Br_5_^2−^, and SeK_5_Cl_5_^2−^. b) Contour maps and c) isosurfaces of the out‐of‐plane component of the induced magnetic field (*B* ^ind^_z_) for all systems analyzed in this work. Units are given in ppm.

Figure 3a shows the vector representation of the magnetically induced current density (**J** ^ind^) in the molecular plane and 1.0 Å above it for SeLi_5_I_5_^2−^, SeNa_5_Cl_5_^2−^, SeNa_5_Br_5_^2−^, and SeK_5_Cl_5_^2−^. The induced currents were confined to localized atomic regions, with no evidence of a global ring current. This localized magnetic behavior was confirmed by the ring current strength (RCS) profiles in Figure S11, which include the integration plane and the corresponding current intensities (in nA/T). In all cases, the total integrated current remained below 1.0 nA/T, consistent with the absence of extended electronic delocalization.

The *z*‐component of the induced magnetic field (*B* ^ind^_z_) was calculated and is shown in Figure 3b,c. The isoline maps indicate that the diatropic response was concentrated around the selenium and halogen atoms, while paratropic regions appeared near the alkali metals. This localized response is also illustrated in the *B* ^ind^_z_ isosurface plots, showing that the diatropic regions surrounded both the selenium and halogen atoms. The intensity of the diatropic field around selenium was comparable to that around the halogens.

These results indicate that the structural stabilization in ppSe species arises mainly from localized electrostatic interactions and geometric constraints rather than aromatic delocalization. The absence of global ring currents and the confinement of the magnetic response to specific atomic regions confirmed their classification as nonaromatic species. AdNDP analysis further revealed localized lone pairs on selenium without involvement in multicenter bonding.

**Figure 4:**
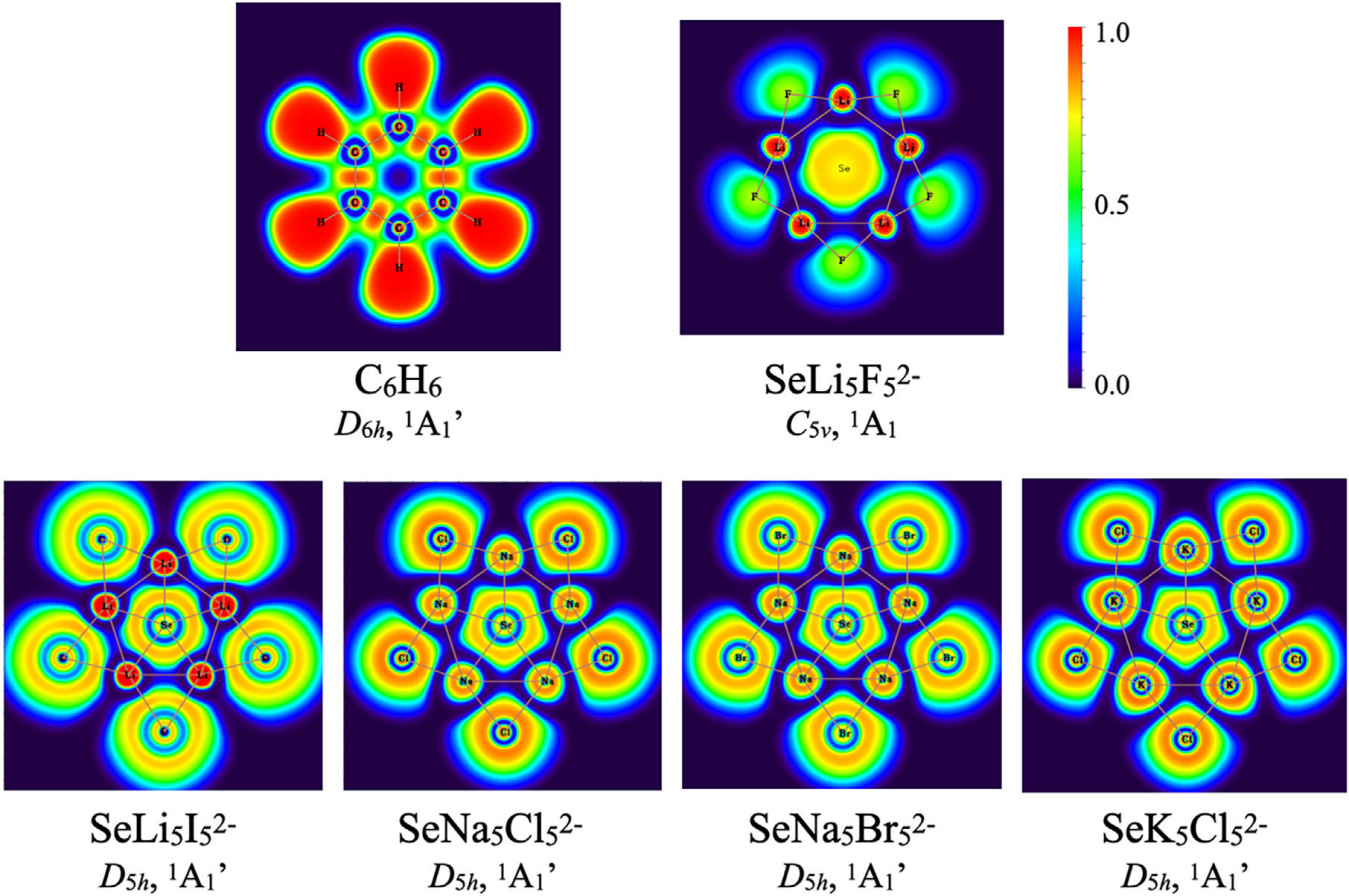
Electron localization function maps for benzene and all ppSe minima computed at PBE0‐D3/def2‐TZVP level.

Although bonding and delocalization had been previously analyzed using several schemes, we have computed ELF maps (Figure 4), as suggested by the reviewer, for benzene and all ppSe minima, including the *C*_5_ *_v_* case (SeLi_2_F_5_^2−^) and the four *D*_5_ *_h_* global minima (SeLi_5_I_5_^2−^, SeNa_5_Cl_5_^2−^, SeNa_5_Br_5_^2−^, SeK_5_Cl_5_^2−^). Benzene exhibits a contiguous, ring‐shaped high‐ELF region characteristic of π delocalization, whereas the ppSe clusters show localized high‐ELF regions on Se, M, and X, with no ring‐connected domains. This ELF topology is consistent across both *C*_5_ *_v_* and *D*_5_ *_h_* structures, ruling out aromatic delocalization and supporting a localized/ionic bonding description under geometric confinement.

The dynamic behavior of SeM_5_X_5_^2−^ clusters (M = Li, Na, K, Rb, and X = F, Cl, Br, and I) was examined using BOMD simulations at 600 K. Under these conditions, all compounds maintained their structural integrity over 30 ps simulations, showing only minor fluctuations, as displayed in the trajectory movies (Movies S1–S11 in the Supporting Information). At elevated temperatures (e.g., 900 K), the increased atomic kinetic energy overcame some energy barriers, leading to structural disruption in certain cases. For example, the star‐like geometry of SeL_i5_F_5_^2−^ was no longer preserved and isomerized into a more disordered configuration after approximately 8 ps of simulation at 900 K.

## 5 Conclusions

Our findings show that ppSe atoms can be stabilized within a specific class of dianionic clusters (SeM_5_X_5_^2−^ where M = Li─Cs and X = F─I) containing alkali metals and halogens arranged to generate a confined, highly symmetric coordination environment. In these species, the bonding pattern is dominated by electrostatic interactions rather than covalent multicenter bonding. The selenium atom retains its lone pairs and does not significantly participate in electron delocalization with the peripheral atoms. As a result, electron density descriptors, electron localization function, and magnetic response analyses indicate the absence of multicenter aromatic stabilization. Planarity results from a delicate balance between geometric constraints and ionic interactions involving the Se center, alkali metals, and halides. These results show that hypercoordination in main‐group elements can be achieved through mechanisms distinct from those associated with aromaticity, providing alternative pathways for designing unusual bonding environments.

## Conflict of Interest

The authors declare no conflict of interest.

## Supporting information



Supporting Information

## Data Availability

The data that support the findings of this study are available in the supplementary material of this article.
