# Supplementary material for: Planar and Pyramidal Pentacoordinate Selenium Atoms
Source: Chemistry. 2025 Nov 24;31(72):e02525. doi: 10.1002/chem.202502525 (PMC12731533; doi:10.1002/chem.202502525)
Supplement: Supplementary file 1 — Supporting Information [file CHEM-31-e02525-s001.docx]

Supporting Information

Planar and Pyramidal Pentacoordinate Selenium Atoms

Luz Diego,^1^ Alejandro Vásquez-Espinal,^2^ Rafael Islas,^3,4^ Gabriel Merino.^*,5^

^1^Doctorado en Fisicoquímica Molecular, Facultad de Ciencias Exactas, Universidad Andres Bello, Av. República 275, Santiago, Chile.

^2^Química y Farmacia, Facultad de Ciencias de la Salud, Universidad Arturo Prat, Iquique 1100000, Chile.

^3^Departamento de Ciencias Químicas, Facultad de Ciencias Exactas, Universidad Andres Bello, Av. República 275, Santiago, Chile.

^4^Centro de Química Teórica & Computacional (CQT&C), Facultad de Ciencias Exactas, Universidad Andres Bello, Av. República 275, Santiago, Chile.

^5^Departamento de Física Aplicada, Centro de Investigación y de Estudios Avanzados, Unidad Mérida, km. 6 Antigua carretera a Progreso. Apdo. Postal 73, Cordemex, Mérida, Yuc., México.

E-mail: gmerino@cinvestav.mx

**Table S1.** Point group (*D*_5_*_h_*, *C*_5_*_v_*, or *C_s_*) and lowest vibrational frequency (cm^-1^, in parentheses) for SeM_5_X_5_^2-^ clusters (M = alkali metals, X = halogens) calculated at the PBE0-D3/def2-TZVP level.

| SeM_5_X_5_^2-^ | | | | |
| --- | --- | --- | --- | --- |
| X  M | F | Cl | Br | I |
| Li | *C*_5_*_v_*, (62.3) | *C*_5_*_v_*, (29.2) | *C*_5_*_v_*, (16.8) | *D*_5_*_h_*, (8.7) |
| Na | *C*_5_*_v_*, (33.8) | *D*_5_*_h_*, (4.6) | *D*_5_*_h_*, (9.7) | *C_s_*, (2.0) |
| K | *C*_5_*_v_*, (15.4) | *D*_5_*_h_*, (3.8) | *C_s_*, (2.3) | *C_s_*, (0.1) |
| Rb | *C*_5_*_v_*, (9.3) | *C_s_*, (3.2) | *C_s_*, (1.7) | *C*_1_, (1.1) |
| Cs | *C_s_*, (2.9) | *C*_1_, (0.7) | *C*_1_, (1.2) | *C_s_*, (0.6) |

**
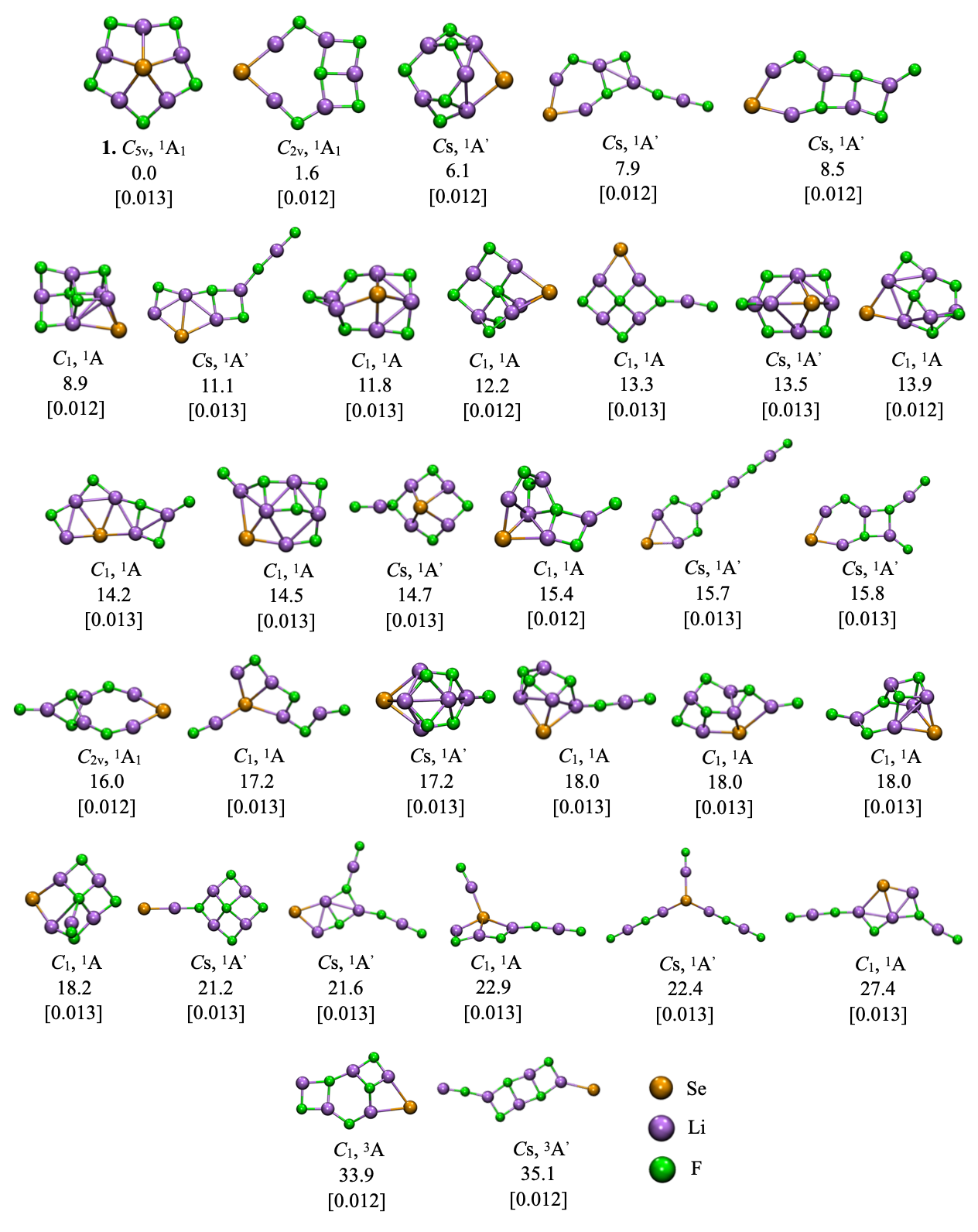
**

**Figure S1.** PBE0-D3/def2-TZVP geometries of the lowest-energy SeLi_5_F_5_^2-^ isomers. Relative energies (kcal/mol) were computed at the CCSD(T)/cc-pVTZ//PBE0-D3/def2-TZVP level, including ZPE corrections. *T*_1_ diagnostic values are given in brackets.

**
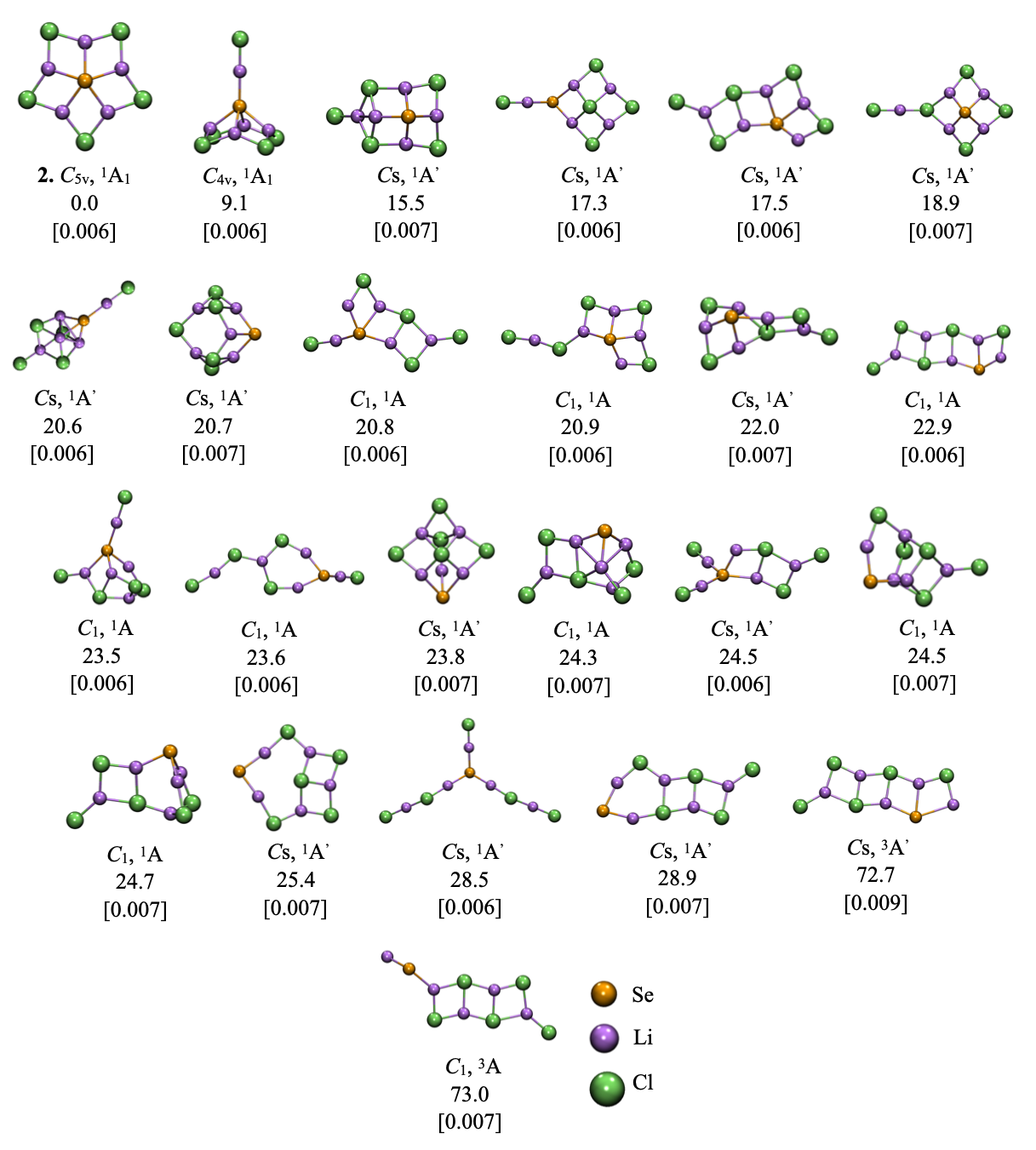
**

**Figure S2.** PBE0-D3/def2-TZVP geometries of the lowest-energy SeLi_5_Cl_5_^2-^ isomers. Relative energies (kcal/mol) were computed at the CCSD(T)/cc-pVTZ//PBE0-D3/def2-TZVP level, including ZPE corrections. *T*_1_ diagnostic values are given in brackets.

**
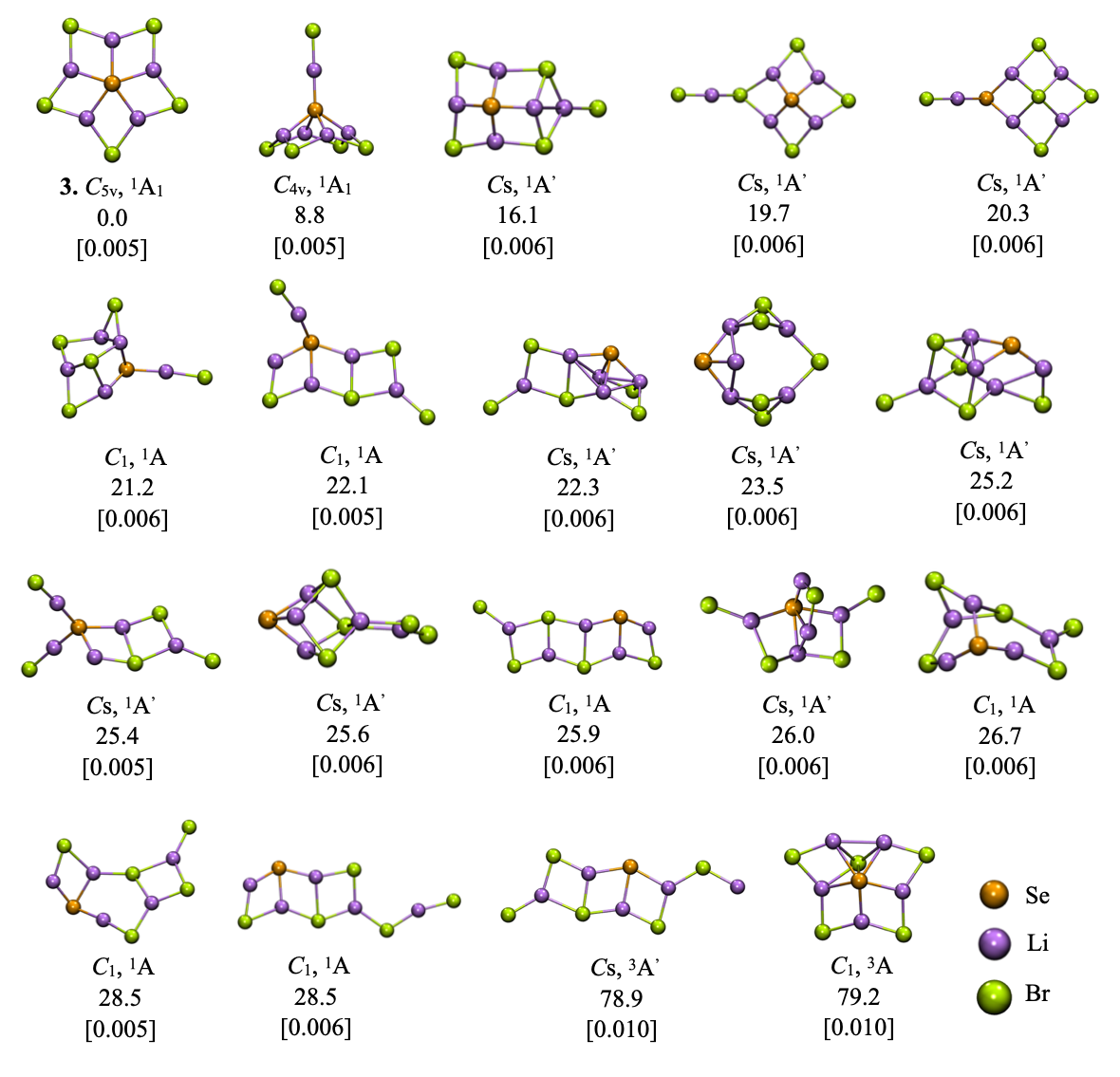
**

**Figure S3.** PBE0-D3/def2-TZVP geometries of the lowest-energy SeLi_5_Br_5_^2-^ isomers. Relative energies (kcal/mol) were computed at the CCSD(T)/cc-pVTZ//PBE0-D3/def2-TZVP level, including ZPE corrections. *T*_1_ diagnostic values are given in brackets.


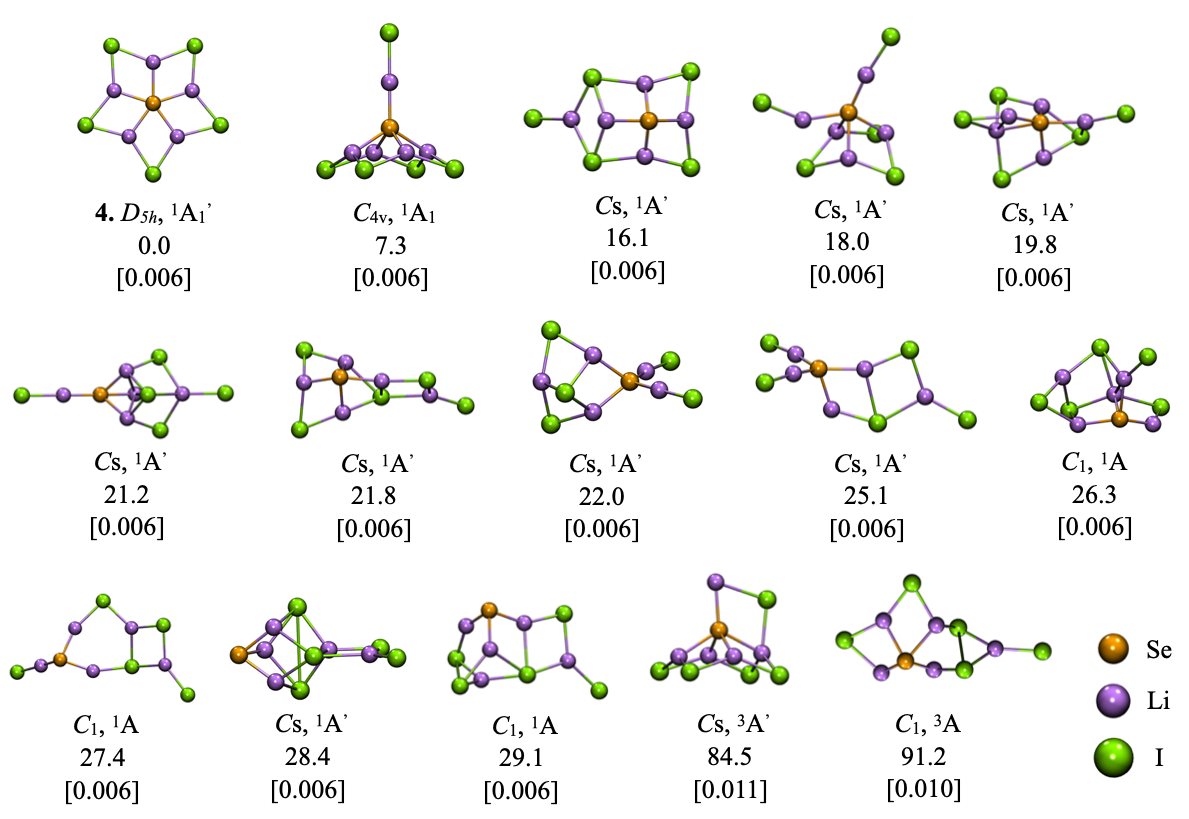


**Figure S4.** PBE0-D3/def2-TZVP geometries of the lowest-energy SeLi_5_I_5_^2-^ isomers. Relative energies (kcal/mol) were computed at the CCSD(T)/cc-pVTZ//PBE0-D3/def2-TZVP level, including ZPE corrections. *T*_1_ diagnostic values are given in brackets.

**
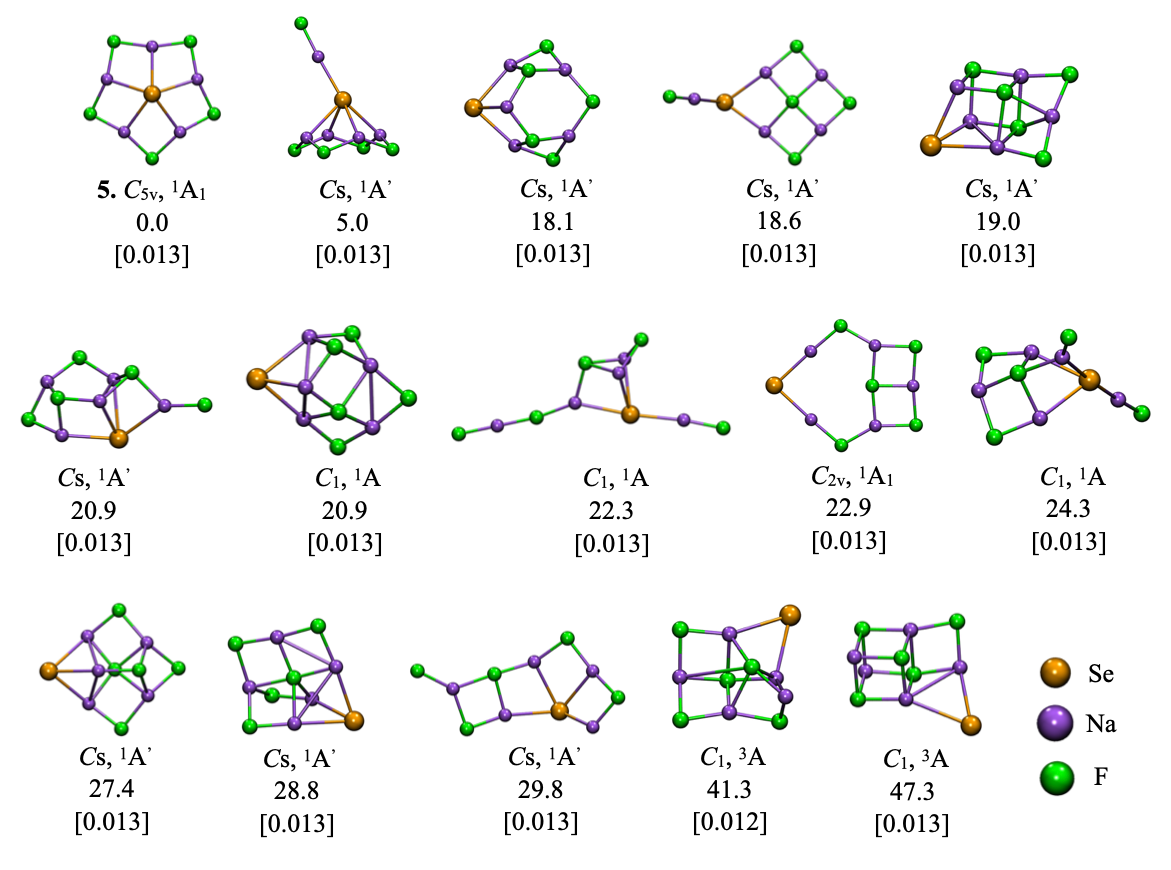
**

**Figure S5.** PBE0-D3/def2-TZVP geometries of the lowest-energy SeNa_5_F_5_^2-^ isomers. Relative energies (kcal/mol) were computed at the CCSD(T)/cc-pVTZ//PBE0-D3/def2-TZVP level, including ZPE corrections. *T*_1_ diagnostic values are given in brackets.


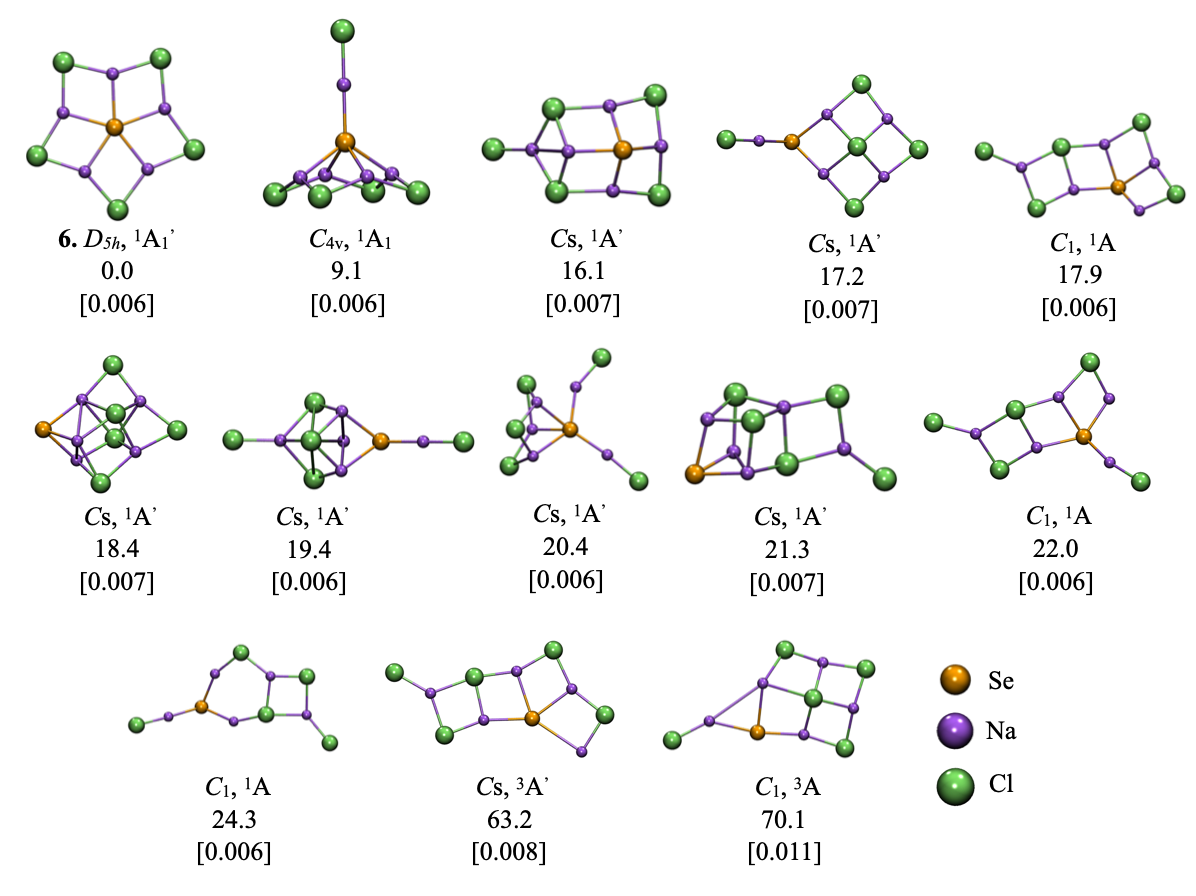


**Figure S6.** PBE0-D3/def2-TZVP geometries of the lowest-energy SeNa_5_Cl_5_^2-^ isomers. Relative energies (kcal/mol) were computed at the CCSD(T)/cc-pVTZ//PBE0-D3/def2-TZVP level, including ZPE corrections. *T*_1_ diagnostic values are given in brackets.

**
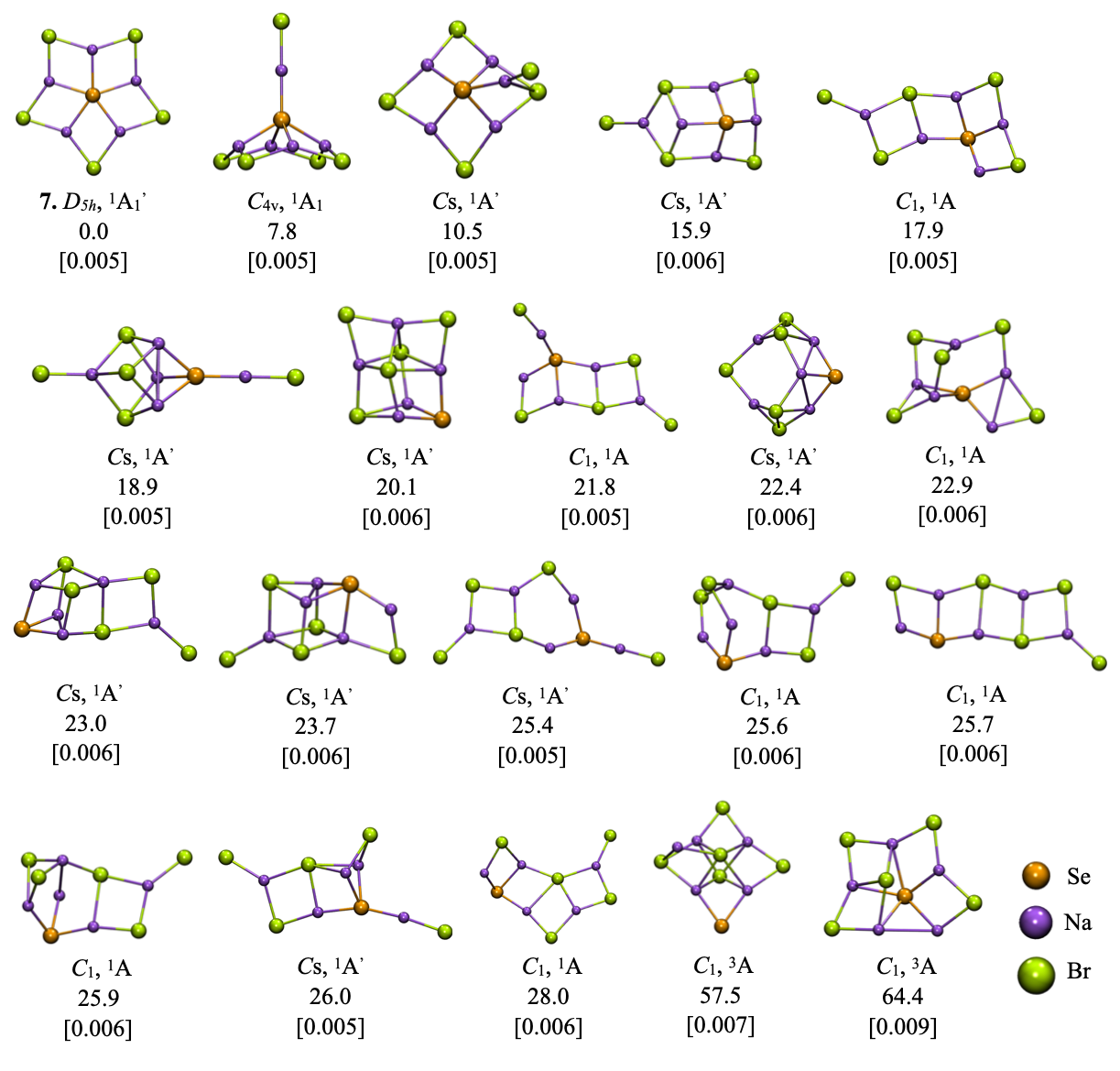
**

**Figure S7.** PBE0-D3/def2-TZVP geometries of the lowest-energy SeNa_5_Br_5_^2-^ isomers. Relative energies (kcal/mol) were computed at the CCSD(T)/cc-pVTZ//PBE0-D3/def2-TZVP level, including ZPE corrections. *T*_1_ diagnostic values are given in brackets.


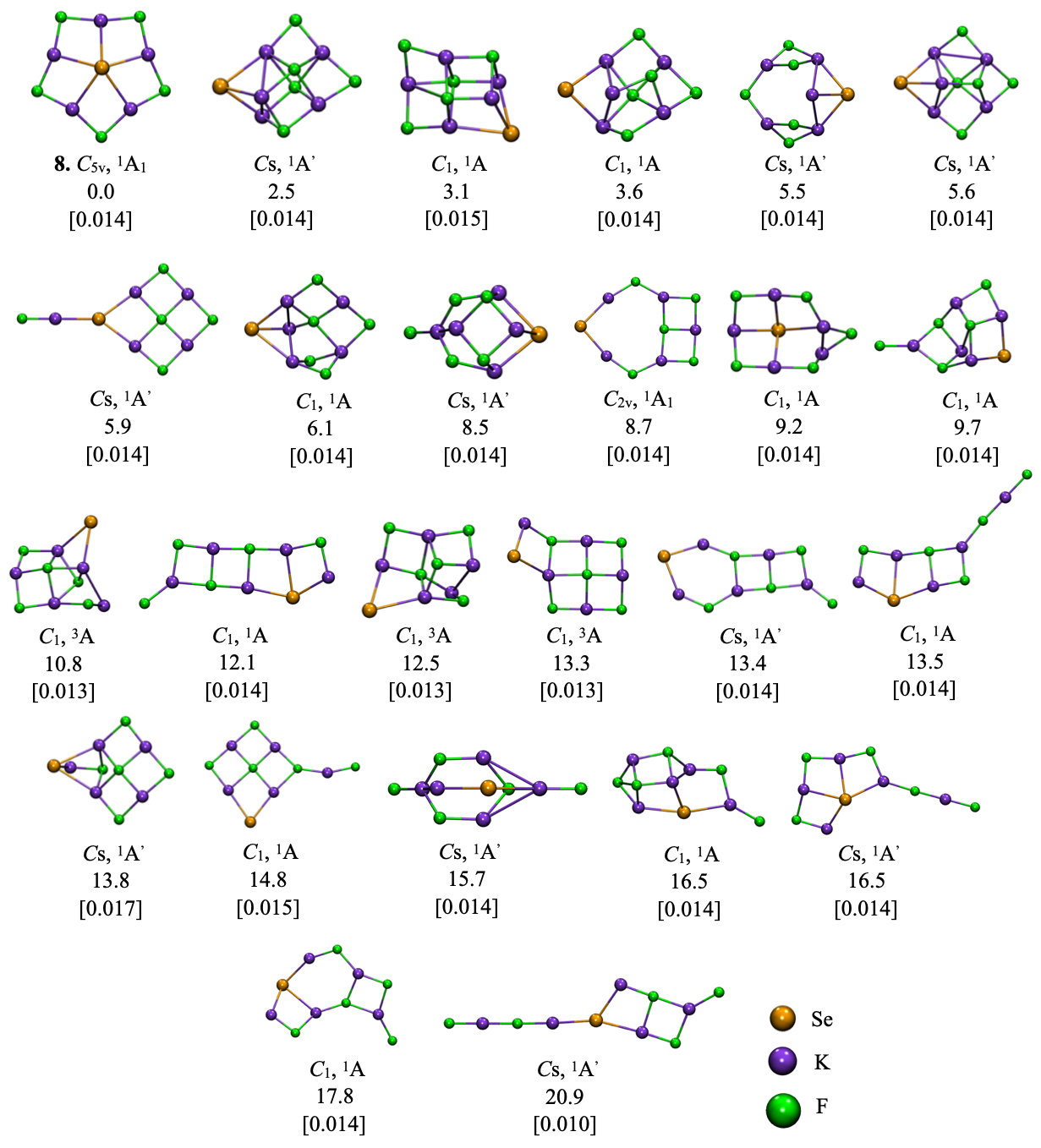


**Figure S8.** PBE0-D3/def2-TZVP geometries of the lowest-energy SeK_5_F_5_^2-^ isomers. Relative energies (kcal/mol) were computed at the CCSD(T)/cc-pVTZ//PBE0-D3/def2-TZVP level, including ZPE corrections. *T*_1_ diagnostic values are given in brackets.

**
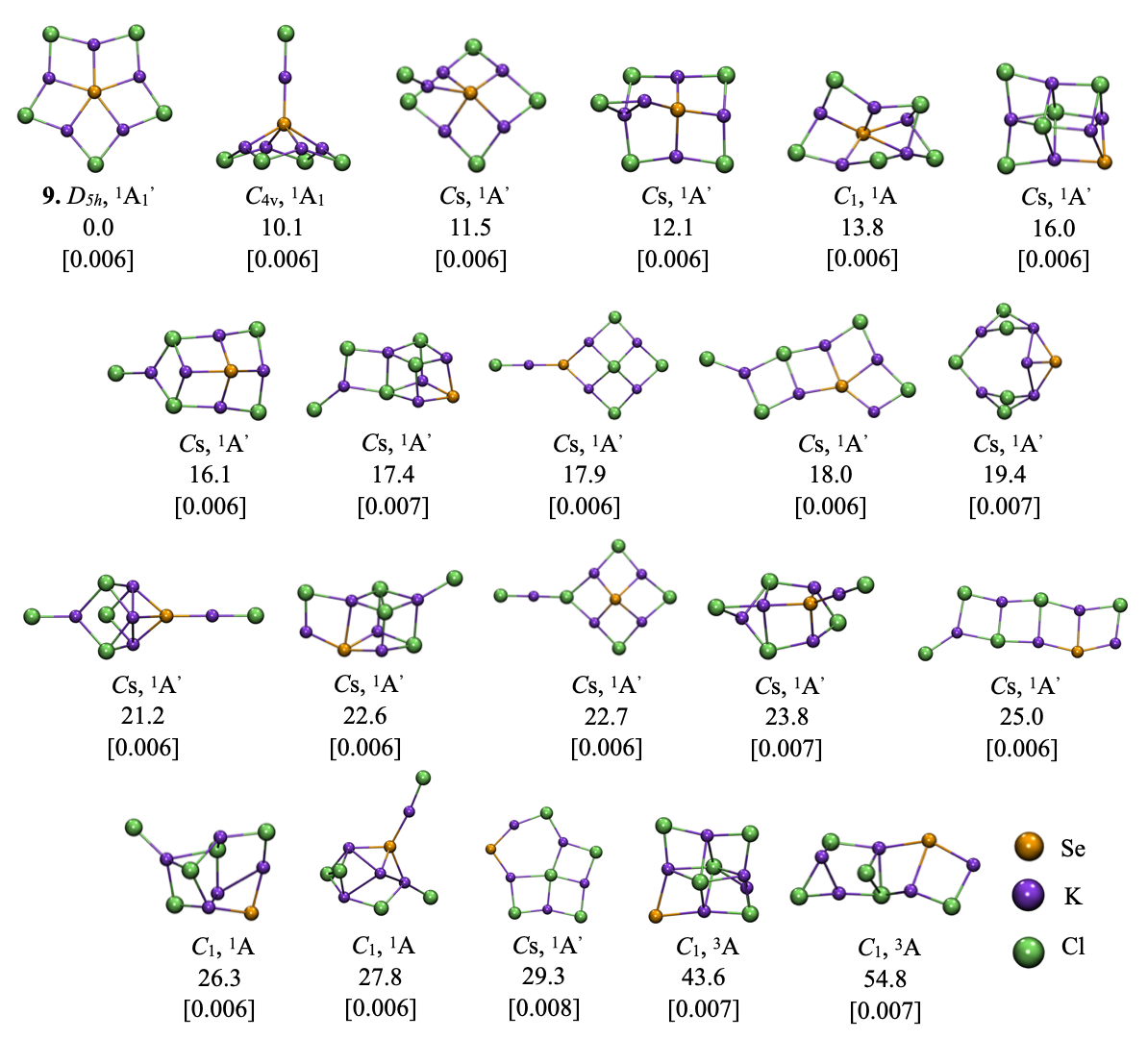
**

**Figure S9.** PBE0-D3/def2-TZVP geometries of the lowest-energy SeK_5_Cl_5_^2-^ isomers. Relative energies (kcal/mol) were computed at the CCSD(T)/cc-pVTZ//PBE0-D3/def2-TZVP level, including ZPE corrections. *T*_1_ diagnostic values are given in brackets.


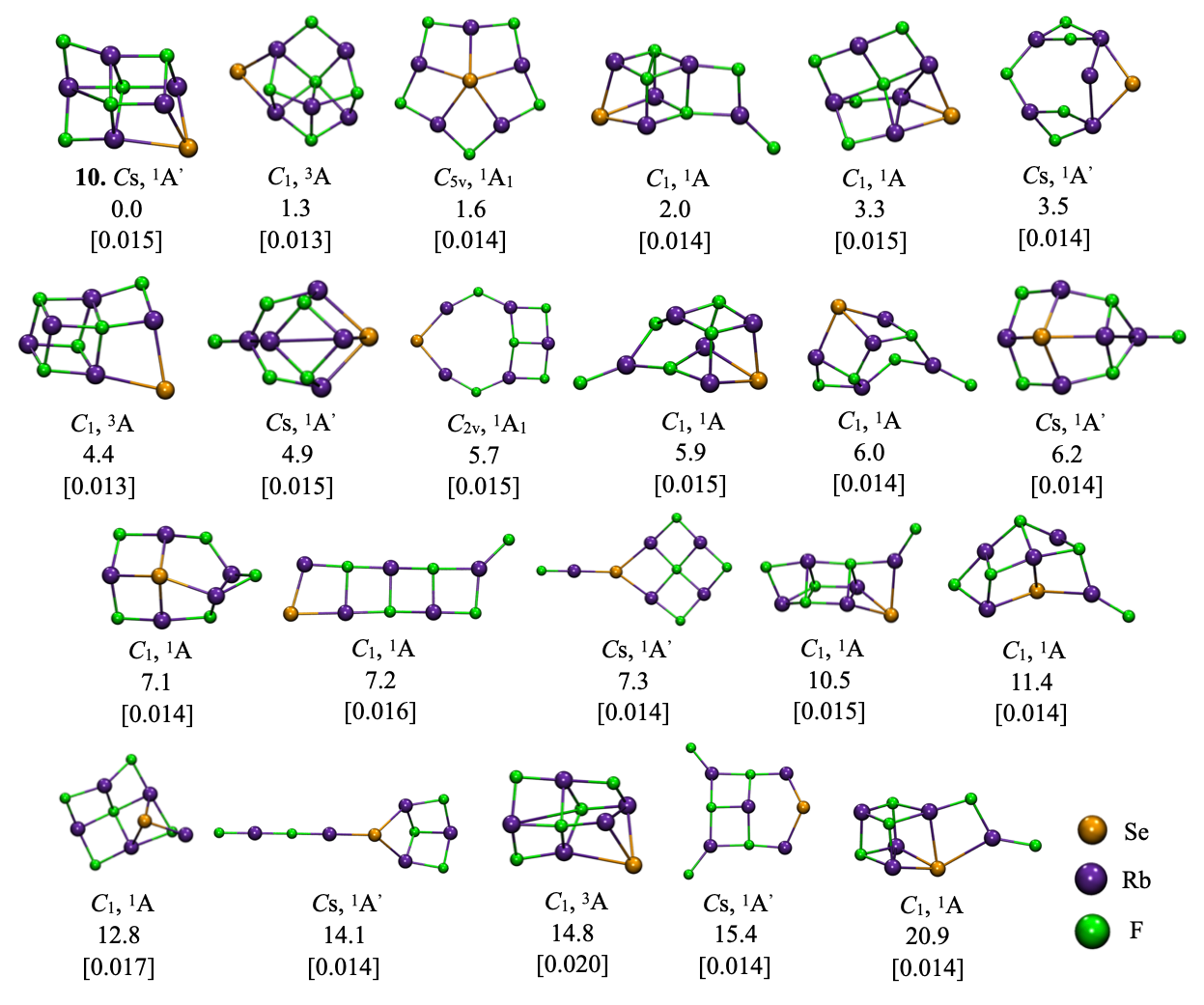


**Figure S10.** PBE0-D3/def2-TZVP geometries of the lowest-energy SeRb_5_F_5_^2-^ isomers. Relative energies (kcal/mol) were computed at the CCSD(T)/cc-pVTZ//PBE0-D3/def2-TZVP level, including ZPE corrections. *T*_1_ diagnostic values are given in brackets.

**Table S2.** Point group (PG), spectroscopic states (State), and lowest vibrational frequency (ν_1_, cm^-1^) for the *D*_5h_ minimum of SeLi_5_I_5_^2-^, SeNa_5_Cl_5_^2-^, SeNa_5_Br_5_^2-^, and SeK_5_Cl_5_^2-^ obtained using various DFT methods with the aug-cc-pVTZ basis set (combined with aug-cc-pVTZ-PP for Se, Br, and I).

| SeLi_5_I_5_^2-^ / aug-cc-pVTZ | | | | | | |
| --- | --- | --- | --- | --- | --- | --- |
|  | PG | State | ν_1_ | *r*_Se-M_ | *r*_M-M_ | *r*_M-E_ |
| TPSS-D3 | *D*_5_*_h_* | ^1^A_1_’ | 9.2 | 2.482 | 2.918 | 2.717 |
| ωB97XD | *D*_5_*_h_* | ^1^A_1_’ | 8.3 | 2.514 | 2.955 | 2.781 |
| M11 | *D*_5_*_h_* | ^1^A_1_’ | 10.2 | 2.505 | 2.945 | 2.723 |
| PBE0-D3 | *D*_5_*_h_* | ^1^A_1_’ | 9.8 | 2.466 | 2.899 | 2.699 |
| M06-2X | *D*_5_*_h_* | ^1^A_1_’ | 11.9 | 2.472 | 2.906 | 2.693 |
| M08-HX | *D*_5_*_h_* | ^1^A_1_’ | 13.3 | 2.493 | 2.931 | 2.701 |
| LC-wPBE-D3 | *D*_5_*_h_* | ^1^A_1_’ | 9.8 | 2.453 | 2.884 | 2.683 |
| SeNa_5_Cl_5_^2-^ / aug-cc-pVTZ | | | | | | |
|  | PG | State | ν_1_ | *r*_Se-M_ | *r*_M-M_ | *r*_M-E_ |
| TPSS-D3 | *D*_5_*_h_* | ^1^A_1_’ | 10.1 | 2.843 | 3.342 | 2.688 |
| ωB97XD | *D*_5_*_h_* | ^1^A_1_’ | 9.9 | 2.833 | 3.331 | 2.702 |
| M11 | *D*_5_*_h_* | ^1^A_1_’ | 15.1 | 2.828 | 3.325 | 2.660 |
| PBE0-D3 | *D*_5_*_h_* | ^1^A_1_’ | 11.0 | 2.819 | 3.314 | 2.664 |
| *M06*-2X | *D*_5_*_h_* | ^1^A_1_’ | 12.4 | 2.793 | 3.283 | 2.630 |
| M08-HX | *D*_5_*_h_* | ^1^A_1_’ | 13.3 | 2.821 | 3.317 | 2.649 |
| LC-wPBE-D3 | *D*_5_*_h_* | ^1^A_1_’ | 15.0 | 2.804 | 3.296 | 2.660 |
| SeNa_5_Br_5_^2-^ / aug-cc-pVTZ | | | | | | |
|  | PG | State | ν_1_ | *r*_Se-M_ | *r*_M-M_ | *r*_M-E_ |
| TPSS-D3 | *D*_5_*_h_* | ^1^A_1_’ | 11.7 | 2.858 | 3.360 | 2.849 |
| ωB97XD | *D*_5_*_h_* | ^1^A_1_’ | 13.2 | 2.850 | 3.351 | 2.865 |
| M11 | *D*_5_*_h_* | ^1^A_1_’ | 12.0 | 2.845 | 3.345 | 2.824 |
| PBE0-D3 | *D*_5_*_h_* | ^1^A_1_’ | 12.1 | 2.834 | 3.332 | 2.820 |
| M06-2X | *D*_5_*_h_* | ^1^A_1_’ | 11.5 | 2.808 | 3.301 | 2.787 |
| M08-HX | *D*_5_*_h_* | ^1^A_1_’ | 11.5 | 2.836 | 3.334 | 2.809 |
| LC-wPBE-D3 | *D*_5_*_h_* | ^1^A_1_’ | 11.3 | 2.818 | 3.313 | 2.812 |
| SeK_5_Cl_5_^2-^ / aug-cc-pVTZ | | | | | | |
|  | PG | State | ν_1_ | *r*_Se-M_ | *r*_M-M_ | *r*_M-E_ |
| TPSS-D3 | *D*_5_*_h_* | ^1^A_1_’ | 12.4 | 3.260 | 3.833 | 3.046 |
| ωB97XD | *D*_5_*_h_* | ^1^A_1_’ | 10.6 | 3.228 | 3.795 | 3.025 |
| M11 | *D*_5_*_h_* | ^1^A_1_’ | 17.5 | 3.229 | 3.796 | 3.002 |
| PBE0-D3 | *D*_5_*_h_* | ^1^A_1_’ | 11.3 | 3.232 | 3.799 | 3.023 |
| M06-2X | *D*_5_*_h_* | ^1^A_1_’ | 11.3 | 3.197 | 3.758 | 2.977 |
| M08-HX | *D*_5_*_h_* | ^1^A_1_’ | 12.0 | 3.216 | 3.781 | 2.990 |
| LC-wPBE-D3 | *D*_5_*_h_* | ^1^A_1_’ | 12.9 | 3.228 | 3.794 | 3.032 |

**Table S3.** Energy decomposition analysis (EDA) at the PBE0-D3/TZ2P-ZORA//PBE0-D3/aug-cc-pVTZ level for SeM_5_X_5_^2-^ clusters with Se^2-^(S,4*s*^2^4*p*^6^) and M_5_X_5_ (S) as fragments. Energy values are given in kcal.mol^-1^.

| System | PG | ᐃ*E*_orb_ | ᐃ*V*_elstat_ | ᐃ*E*_disp_ | ᐃ*E*_Pauli_ | ᐃ*E*_int_ | %ᐃ*E*_orb_ | %ᐃ*V*_elstat_ | %ᐃ*E*_disp_ |
| --- | --- | --- | --- | --- | --- | --- | --- | --- | --- |
| SeLi_5_F_5_^2-^ | *D*_5_*_h_* | -73.1 | -299.35 | -3.46 | 189.71 | -186.2 | 19.4 | 79.6 | 0.9 |
| SeLi_5_F_5_^2-^ | *C*_5_*_v_* | -60.94 | -252.4 | -3.28 | 102.78 | -213.84 | 19.2 | 79.7 | 1.0 |
| SeLi_5_Cl_5_^2-^ | *D*_5_*_h_* | -82.58 | -327.93 | -4.83 | 146.92 | -268.42 | 19.9 | 79.0 | 1.2 |
| SeLi_5_Cl_5_^2-^ | *C*_5_*_v_* | -85.22 | -315.92 | -4.71 | 130.46 | -275.38 | 21.0 | 77.8 | 1.2 |
| SeLi_5_Br_5_^2-^ | *D*_5_*_h_* | -89.94 | -329.17 | -5.12 | 140.59 | -283.65 | 21.2 | 77.6 | 1.2 |
| SeLi_5_Br_5_^2-^ | *C*_5_*_v_* | -91.47 | -325.16 | -5.08 | 135.86 | -285.84 | 21.7 | 77.1 | 1.2 |
| SeLi_5_I_5_^2-^ | *D*_5_*_h_* | -99.79 | -331.96 | -5.51 | 136.51 | -300.75 | 22.8 | 75.9 | 1.3 |
| SeNa_5_F_5_^2-^ | *D*_5_*_h_* | -34.29 | -307.86 | -3.42 | 142.23 | -203.33 | 9.9 | 89.1 | 1.0 |
| SeNa_5_F_5_^2-^ | *C*_5_*_v_* | -35.98 | -277.85 | -3.26 | 96.6 | -220.48 | 11.3 | 87.6 | 1.0 |
| SeNa_5_Cl_5_^2-^ | *D*_5_*_h_* | -46.22 | -330.97 | -4.23 | 114.83 | -266.6 | 12.1 | 86.8 | 1.1 |
| SeNa_5_Br_5_^2-^ | *D*_5_*_h_* | -54.05 | -330.84 | -4.43 | 111 | -278.31 | 13.9 | 85.0 | 1.1 |
| SeK_5_F_5_^2-^ | *D*_5_*_h_* | -42.96 | -279.78 | -3.44 | 135.19 | -191 | 13.2 | 85.8 | 1.1 |
| SeK_5_F_5_^2-^ | *C*_5_*_v_* | -40.63 | -259.55 | -3.34 | 107.02 | -196.5 | 13.4 | 85.5 | 1.1 |
| SeK_5_Cl_5_^2-^ | *D*_5_*_h_* | -47.9 | -314.1 | -3.84 | 112.01 | -253.81 | 13.1 | 85.9 | 1.0 |


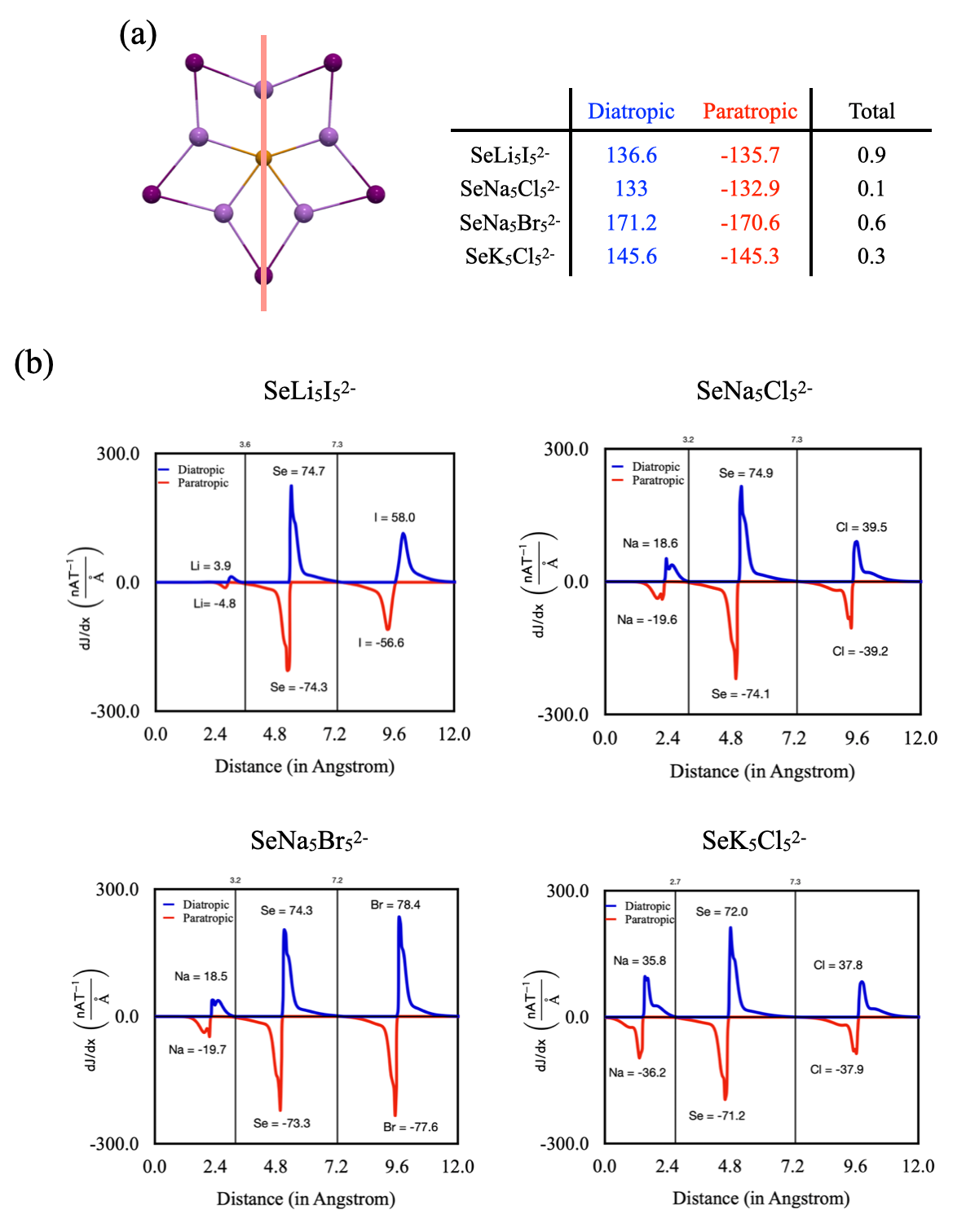


**Figure S11.** (a) Integration plane used for the calculation, showing the diatropic, paratropic, and total current intensities (nA/T) (b) Ring current strength (RCS) profiles (nA/T) along the integration plane for the global minima of the ppSe systems SeLi_5_I_5_^2-^, SeNa_5_Cl_5_^2-^, SeNa_5_Br_5_^2-^, and SeK_5_Cl_5_^2-^. Calculations were performed at the BHandHLYP/def2-TZVP level.

**Coordinates of PBE0-D3/def2-TZVP**

SeLi_5_F_5_^2-^, *C*_5_*_v_*

Se 0.000000000 -0.000000192 1.137173161

Li 0.000000000 2.130942896 -0.330824712

Li 2.026647000 0.658497896 -0.330824800

Li -2.026647000 0.658497896 -0.330824800

Li 1.252537000 -1.723969104 -0.330824943

Li -1.252537000 -1.723969104 -0.330824943

F 1.699082000 2.338585926 -0.834528699

F -1.699082000 2.338585926 -0.834528699

F 2.749173000 -0.893260074 -0.834528893

F 0.000000000 -2.890651074 -0.834529013

F -2.749173000 -0.893260074 -0.834528893

SeLi_5_Cl_5_^2-^, *C*_5_*_v_*

Li 0.000000000 2.349901000 0.034581000

Li -2.234889000 0.726159000 0.034581000

Cl 2.172067000 2.989594000 -0.353989000

Li 2.234889000 0.726159000 0.034581000

Cl -2.172067000 2.989594000 -0.353989000

Li 1.381237000 -1.901110000 0.034581000

Cl 3.514479000 -1.141923000 -0.353989000

Cl 0.000000000 -3.695341000 -0.353989000

Se 0.000000000 0.000000000 0.869716000

Li -1.381237000 -1.901110000 0.034581000

Cl -3.514479000 -1.141923000 -0.353989000

SeLi_5_Br_5_^2-^, *C*_5_*_v_*

Br 0.000000000 -3.960985000 -0.134612000

Se 0.000000000 0.000000000 0.640991000

Br -2.328209000 3.204505000 -0.134612000

Li 0.000000000 2.419869000 0.117559000

Li -1.422363000 -1.957715000 0.117559000

Li 1.422363000 -1.957715000 0.117559000

Br 3.767121000 -1.224012000 -0.134612000

Li 2.301432000 0.747781000 0.117559000

Br 2.328209000 3.204505000 -0.134612000

Li -2.301432000 0.747781000 0.117559000

Br -3.767121000 -1.224012000 -0.134612000

SeLi_5_I_5_^2-^, *D*_5_*_h_*

I 2.508474000 3.452619000 0.000000000

Se 0.000000000 0.000000000 0.000000000

I -4.058797000 -1.318783000 0.000000000

I -2.508474000 3.452619000 0.000000000

Li 0.000000000 2.472549000 0.000000000

I 4.058797000 -1.318783000 0.000000000

I 0.000000000 -4.267671000 0.000000000

Li -2.351534000 0.764060000 0.000000000

Li 2.351534000 0.764060000 0.000000000

Li 1.453328000 -2.000334000 0.000000000

Li -1.453328000 -2.000334000 0.000000000

SeNa_5_F_5_^2-^, *C*_5_*_v_*

F 2.073800000 2.854341000 -0.660523000

F 0.000000000 -3.528159000 -0.660523000

Na 0.000000000 2.575654000 -0.172024000

Na 2.449593000 0.795921000 -0.172024000

Se 0.000000000 0.000000000 1.152495000

F -2.073800000 2.854341000 -0.660523000

Na -1.513932000 -2.083748000 -0.172024000

F 3.355479000 -1.090261000 -0.660523000

Na 1.513932000 -2.083748000 -0.172024000

Na -2.449593000 0.795921000 -0.172024000

F -3.355479000 -1.090261000 -0.660523000

SeNa_5_Cl_5_^2-^, *D*_5_*_h_*

Na 0.000000000 2.815154000 0.000000000

Na 2.677371000 0.869930000 0.000000000

Cl 2.557767000 3.520464000 0.000000000

Cl 4.138553000 -1.344698000 0.000000000

Na 1.654706000 -2.277507000 0.000000000

Cl 0.000000000 -4.351533000 0.000000000

Na -1.654706000 -2.277507000 0.000000000

Cl -4.138553000 -1.344698000 0.000000000

Se 0.000000000 0.000000000 0.000000000

Na -2.677371000 0.869930000 0.000000000

Cl -2.557767000 3.520464000 0.000000000

SeNa_5_Br_5_^2-^, *D*_5_*_h_*

Na 0.000000000 2.832752000 0.000000000

Na -2.694107000 0.875369000 0.000000000

Na 1.665050000 -2.291745000 0.000000000

Br 4.336321000 -1.408956000 0.000000000

Na 2.694107000 0.875369000 0.000000000

Br 2.679994000 3.688695000 0.000000000

Br -2.679994000 3.688695000 0.000000000

Na -1.665050000 -2.291745000 0.000000000

Br -4.336321000 -1.408956000 0.000000000

Br 0.000000000 -4.559478000 0.000000000

Se 0.000000000 0.000000000 0.000000000

SeK_5_F_5_^2-^, *C*_5_*_v_*

F 2.475512000 -3.327859000 -0.596038000

F -3.958723000 1.236111000 -0.596615000

K 2.924363000 -0.913780000 -0.096515000

K 0.034839000 -3.061486000 -0.100016000

Se -0.000055000 0.000362000 1.062530000

F 3.929742000 1.325614000 -0.596144000

K -1.828674000 2.457361000 -0.098581000

F -2.399122000 -3.383435000 -0.595920000

K -2.902954000 -0.979967000 -0.096435000

K 1.772359000 2.498289000 -0.098327000

F -0.047057000 4.147320000 -0.595104000

SeK_5_Cl_5_^2-^, *D*_5_*_h_*

K 0.000000000 3.251136000 0.000000000

Cl -4.735381000 -1.538619000 0.000000000

Se 0.000000000 0.000000000 0.000000000

Cl -2.926627000 4.028156000 0.000000000

Cl 4.735381000 -1.538619000 0.000000000

Cl 0.000000000 -4.979074000 0.000000000

K 1.910970000 -2.630224000 0.000000000

K 3.092014000 1.004656000 0.000000000

Cl 2.926627000 4.028156000 0.000000000

K -1.910970000 -2.630224000 0.000000000

K -3.092014000 1.004656000 0.000000000

SeRb_5_F_5_^2-^, *C*_5_*_v_*

Se 0.000000000 0.000000000 1.008640000

Rb 0.000000000 3.287679000 -0.050865000

Rb 3.126769000 1.015949000 -0.050865000

Rb -3.126769000 1.015949000 -0.050865000

Rb 1.932449000 -2.659788000 -0.050865000

Rb -1.932449000 -2.659788000 -0.050865000

F 2.608406000 3.590163000 -0.552972000

F -2.608406000 3.590163000 -0.552972000

F 4.220490000 -1.371320000 -0.552972000

F 0.000000000 -4.437686000 -0.552972000

F -4.220490000 -1.371320000 -0.552972000
